# Uncovering the role of wheat magnesium transporter family genes in abiotic responses

**DOI:** 10.3389/fpls.2023.1078299

**Published:** 2023-02-09

**Authors:** Yanhong Tang, Xiaoyue Yang, Han Li, Yating Shuai, Wang Chen, Dongfang Ma, Zhichuang Lü

**Affiliations:** ^1^ MARA Key Laboratory of Sustainable Crop Production in the Middle Reaches of the Yangtze River (Co-construction by Ministry and Province)/Engineering Research Center of Ecology and Agricultural Use of Wetland, Ministry of Education/College of Agriculture, Yangtze University, Jingzhou, China; ^2^ State Key Laboratory for Biology of Plant Diseases and Insect Pests, Institute of Plant Protection, Chinese Academy of Agricultural Sciences, Beijing, China

**Keywords:** TaMGT, gene structure, abiotic stresses, gene expression, fluorescence quantitative PCR, climate-resilience

## Abstract

**Background:**

The CorA / MGT / MRS2 family proteins are an important group of magnesium transporter proteins that maintain magnesium ion homeostasis in plant cells. However, little is known about the MGT functions in wheat.

**Methods:**

The known MGT sequences were used as queries to BlastP against wheat genome IWGSC RefSeq v2.1 assembly (E-value <10–5). Chromosome localization information for each *TaMGT* gene was obtained from the GFF3 file of the wheat genome data (IWGSCv2.1).The sequence of 1500 bp upstream of the *TaMGT* genes was extracted from the wheat genome data. The cis-elements were analyzed using PlantCARE online tool.

**Result:**

A total of 24 *MGT* genes were identified on 18 chromosomes of wheat. After functional domain analysis, only *TaMGT1A*, *TaMGT1B*, and *TaMGT1D* had GMN mutations to AMN, while all the other genes had conserved GMN tripeptide motifs. Expression profiling showed that the *TaMGT* genes were differentially expressed under different stresses and at different growth and development stages. The expression levels of *TaMGT4B* and *TaMGT4A* were significantly up-regulated in cold damage. In addition, qRT-PCR results also confirmed that these *TaMGT* genes are involved in the wheat abiotic stress responses.

**Conclusion:**

In conclusion, The results of our research provide a theoretical basis for further research on the function of *TaMGT* gene family in wheat.

## 1 Introduction

Magnesium (Mg^2+^) is an important macronutrient (Williams and Salt, 2009). As the most abundant divalent cation in living plant cells, Mg^2+^ is involved in chloroplast synthesis, regulation of osmotic pressure, and intracellular enzyme activity (Shaul, 2002; Williams and Salt, 2009; Guo et al., 2016). In addition, Mg^2+^ is essential for the synthesis of proteins and nucleic acids and maintains the cation-anion balance in cell (Marschner and Marschner, 2012). Moreover, magnesium deficiency can adversely affect plant cells, like reducing macromolecular synthesis, photosynthetic capacity, and plant growth (Hermans et al., 2005; Tang et al., 2012; Yang et al., 2012; Peng et al., 2015; Farhat et al., 2016; Li et al., 2016). Therefore, maintaining the balance and stability of Mg is crucial in plants (Shaul, 2002; Kobayashi and Tanoi, 2015).

Magnesium transporter proteins (MGTs), also known as Magnesium Transporter MRS2, play a critical role in maintaining Mg homeostasis in plant cells (Li et al., 2017). *ZmMGT10* has the ability to transport Mg under conditions of Mg deficiency in Maize (Li et al., 2016). The transgenic Arabidopsis plants with *ZmMGT10* overexpression exhibited vigorous growth, such as larger plant size, longer root length, higher fresh weight and increased chlorophyll content compared with the wild-type plants (Li et al., 2017). Moreover, *AtMGT7* can maintain normal growth and development in Arabidopsis under low Mg conditions (Mao et al., 2008).

Among the magnesium transporters, the CorA-type magnesium transporter is the most widely studied magnesium transporter protein. This type of magnesium transporter was originally identified in *Salmonella typhimurium*, and its mutants exhibit resistance to Co^2+^ growth inhibition (Silver, 1969). CorA is a funnel-shaped homopentamer with two transmembrane structural domains per monomer, the first of which forms the ion conduction pathway. Mg^2+^ transport firstly involves the binding of a cation to an extracellular binding loop connecting the transmembrane structural domains (Maguire, 2006). These proteins have a variable conserved N-terminal hydrophilic domain of about 260 amino acids and a fairly conserved hydrophobic domain of 55 amino acids (Maguire, 2006). The MPEL sequence and the YGMNF sequence are the most characteristic features of CorA proteins, both of which are located in the loop between the transmembrane helices. Among them, the (Gly-Met-Asn) GMN sequence is essential for the protein function (Palombo et al., 2013).

CorA-type Mg transport proteins mediate the influx and efflux of Mg^2+^ (Franken et al., 2022; Papp-Wallace and Maguire, 2008). CorA homologous proteins have been identified in yeast, animals and plants (Knoop et al., 2005). In yeast, the ALR1 family confers Al^3+^ tolerance and encodes the major plasma membrane Mg^2+^ uptake system (Liu et al., 2002). Overexpression of *AtMGT1* in tobacco plants increases Mg concentration and confers tolerance to low Mg environments (Deng et al., 2006).

Mutations in the GMN motif may eliminate Mg^2+^ transport, but the naturally occurring variants GVN and GIN may be associated with the transport of other divalent cations (Eshaghi et al., 2006). The GMN motif was changed to AMN in ZmMGT6 in maize, and in functional complementation experiments using *Salmonella typhimurium* mutant MM281, *ZmMGT6* was shown to rescue the susceptibility of Mg^2+^ in *Salmonella typhimurium mutant* MM281, but its complementation efficacy was lower than that of other ZmMGTs containing GMN motifs (Li et al., 2016). In rice, the GMN motif was changed to AMN in *OsMRS2-4* and *OsMRS2-5* and to GIN in *OsMRS2-8*, and in functional complementation experiments using Yeast mutant CM66, *OsMRS2-4*, *-5*, and *-8* did not observe complementation ability, but other *OsMRS2* genes had obvious complementation ability (Saito et al., 2013). These phenomena suggest that GMN motif mutations affected gene functions. Overall, these features are important markers of Mg^2+^ transporters. (Szegedy and Maguire, 1999; Knoop et al., 2005).

In plants, MGT proteins have been reported in *Arabidopsis thaliana* (Schock et al., 2000), maize (*Zea mays*) (Li et al., 2016), and rice (*Oryza sativa*) (Saito et al., 2013). Recently, it has been studied in tomato (*Solanum lycopersicum*) (Regon et al., 2019), sugarcane (*Saccharum spontaneum*) (Wang et al., 2019), pear (*Pyrus bretschneideri*) (Zhao et al., 2018), citrus (*Poncirus trifoliata*) (Liu et al., 2019), *Vitis vinifera* (Ge et al, 2022), *Citrullus lanatus* and *Cucumis sativus* (Heidari et al, 2022), *Theobroma cacao*, *Corchorus capsularis*, and *Gossypium hirsutum* (Heidari et al, 2021), and *Triticum turgidum* and *Camelina sativa* (Faraji et al, 2021). Studies have shown that Mg transporter proteins have different functions. The pear *PbrMGT7* gene mediates Mg transport between mitochondria and the cytoplasmic matrix (Zhao et al., 2018). The sugarcane *MTG6* gene is the main MGT that maintains the concentration of Mg in chlorophyll and transporting Mg ions into the chloroplast stroma, and *MGT10* transports Mg from roots to leaves over long distances (Wang et al., 2019). Banana *MaMRS2-5* and *MaMRS2-7* are involved in the uptake and transport of Mg and its partitioning between different tissues (Tong et al., 2020). The Arabidopsis *AtMGT5* and *AtMGT9* genes play critical roles in Mg supply during pollen mitosis and pollen in formation (Drummond et al., 2006; Chen et al., 2009; Li et al., 2015). In the Malvaceae family, MGTs appear to be involved in various pathways that control plant growth and development and respond to adverse conditions (Heidari et al, 2021). At the same time, MGTs can also affect the biosynthesis of phytohormones related to stresses by regulating Mg^2+^ concentration, thereby enhancing plant stress resistance (Guo et al., 2014).

Wheat (*Triticum aestivum*) is an important food crop worldwide, and Mg^2+^ deficiency may affect chlorophyll synthesis, multiple enzymes activation, photosynthesis, and partitioning and utilization of photoassimilates, which may inhibit the growth and development of wheat and further lead to yield loss (Shaul, 2002; Cakmak and Yazici, 2010). To date, studies on Mg^2+^ transporters in wheat have not been reported. In this study, the Mg^2+^ transporter genes in wheat were identified, their physicochemical properties, chromosomal location and gene structure were systematically analyzed, and their responses to Mg deficiency, Al stress, and abscisic acid treatment were investigated. The present study will provide a basis for further revealing the biological functions of *MGT* genes in wheat.

## 2 Materials and methods

### 2.1 Identification of *MGT* genes in wheat

In this experiment, 10 MGT protein sequences from Arabidopsis, 9 from rice, and 12 from maize were obtained from the Arabidopsis genome database (https://www.arabidopsis.org/), the rice genome database (http://Rice.plantbiology.msu.edu), and the maize genome database (https://www.maizegdb.org/), respectively. The known MGT sequences were used as queries to BlastP against wheat genome IWGSC RefSeq v2.1 assembly (E-value <10^-5^). Duplicates and mismatched candidate proteins were removed. The conserved protein domains were screened through the Pfam database (http://pfam.xfam.org/) (Finn et al., 2006) and the SMART database (http://smart.embl-heidelberg.de/) (Letunic et al., 2004). TaMGT family members were finally determined after excluding the sequences that did not contain the CorA type domain. Subcellular localization prediction of TaMGTs was performed *via* the Plant-mPLoc online tool (http://www.csbio.sjtu.edu.cn/bioinf/plant-multi/). Potential TM regions in each TaMGT protein were predicted using TMHM Server v2.0 (http://www.cbs.dtu.dk/services/TMHMM/) (Krogh et al., 2001). Protein length, average molecular weight, isoelectric point (pI), instability index, and mean hydrophilicity value (GRAVY) were predicted by ExPASy Server10 (SIB Bioinformatics Resource Portal, https://prosite.expasy.org/PS50011).

### 2.2 Construction of Phylogenetic tree, gene structure and protein motif analysis

The protein sequences of 10 AtMGTs, 9 OsMGTs, 12 ZmMGTs, and TaMGTs identified in present study were collected. Multiple sequence alignment was performed using ClustalW2 (Thompson et al., 1994). The TM structural domain and conserved GMN motifs were annotated using DNAMAN software (Zhao et al., 2012). The phylogenetic tree was constructed using MEGA7.0 software (version 7.0, Mega Limited, Auckland, New Zealand) using Neighbor-joining (NJ) method (Kumar et al., 2016) with the Bootstrap value setting as 1000. Furthermore, the phylogenetic tree was modified *via* the online tool iTOL (version 3.2.317, http://itol.embl.de) (Letunic and Bork, 2019).

To study the gene structures of *TaMGTs* genes, the genome annotation information of TaMGTs was obtained from wheat genome database IWGSC V2.1. And the gene structures were visualized by TBtools (Chen et al., 2020). The conserved protein motifs were analyzed by MEME Suite 5.1.1, with the motif number setting as 20 and other parameters with default values.

### 2.3 Chromosomal localization and gene duplication events

Chromosome localization information for each *TaMGT* gene was obtained from the GFF3 file of the wheat genome data (IWGSCv2.1). The chromosome distribution map of the *TaMGT* gene was subsequently generated using MapInspect software. MCScanX software was used to analyze *TaMGT* gene duplication events in wheat and the homology of *MGT* genes between wheat and other selected species(Wang et al., 2012). Duplicate gene pairs among *TaMGT* members were identified and visualized using the R package “circlize”.

### 2.4 Cis-elements and transcriptome expression analysis

To identify cis-regulatory elements in the promoter regions, the sequence of 1500 bp upstream of the *TaMGT* genes was extracted from the wheat genome data. The cis-elements were analyzed using PlantCARE online tool (Lescot et al., 2002). The data was collected and visualized using the R package “pheatmap”.

The RNA-seq data of *TaMGTs* was downloaded through the NCBI SRA database (The SRA numbers are detailed in **Table S3**)(Ramírez-González et al., 2018) and the raw data was aligned to the wheat reference genome by Hisat2 (Kim et al., 2015). The genes were then assembled by cufflinks to detect the expression levels of *TaMGTs* (Fragments per Kilobase Million of exon model per Million mapped fragments, FPKM) (Trapnell et al., 2012). Convert all the transcription data through log2, P<0.05. Heatmaps were created using the R package “pheatmap” to show the expression patterns of *TaMGT* genes under different conditions.

### 2.5 Plant materials and stress treatments

Seeds of the hexaploid wheat variety “Yangmai 158” were surface sterilized in 0.5% (w/v) sodium hypochlorite for 15 min. After germinated in a greenhouse at 25°C for 3 d, the seedlings were transferred to Hoagland solution (pH5.8) and were grown under standard greenhouse conditions. The parameters were set as follows: 16h/25°C and 8h/20°C diurnal cycle, 70% relative humidity, and 300 mmolm-2s-1 strong luminosity. The nutrient solution was replaced every two days.

For Mg^2+^ deficiency treatment, wheat seedlings at two leaves with a bud stage were transferred to Hogeland solution with MgSO_4_-7H_2_O deficient. For aluminium chloride stress treatment, wheat seedlings were transferred to a Hoagland solution supplemented with 60uM aluminium chloride (pH4.5). The roots and leaves of treated seedlings were collected at different time points (6h, 12h, 24h, 48h, and 72h after treatment). For hormone treatment, seedlings were treated with 100 mmol/L abscisic acid. Roots and leaves were sampled at 6h, 12h, 24h, and 48h after treatment. Untreated wheat seedlings were used as control. Three biological replicates were set up for each treatment, with each replicate including three technical replicates. The collected samples were immediately frozen in liquid nitrogen and stored at -80°C for subsequent RNA extraction.

### 2.6 Real-time quantitative RT-PCR analysis

The relative expression levels of *TaMGT* genes in roots and leaves of wheat under Mg^2+^ deficiency (-Mg^2+^), aluminum stress (+Al), and abscisic acid (+ABA) treatments were analyzed by qRT-PCR. Total RNA was extracted from roots and leaves using TRIzol reagent (Life, USA). cDNA was reverse transcribed using the HiScript II Reverse Transcriptase kit (Vazyme, Nanjing, China).Three biological replicates were performed for each sample, with three technical replicates repeated each. The qPCR primers used in present study were listed in **Supplementary Table 1**. The *Ta2291* gene, which was expressed stably under various conditions, was used as the internal reference gene (Paolacci et al., 2009). The expression levels of *TaMGT* genes were calculated using 2^−ΔΔCt^ method (Livak and Schmittgen, 2001).

## 3 Results

### 3.1 Identification of *MGT* genes in wheat

**Figure 1:**
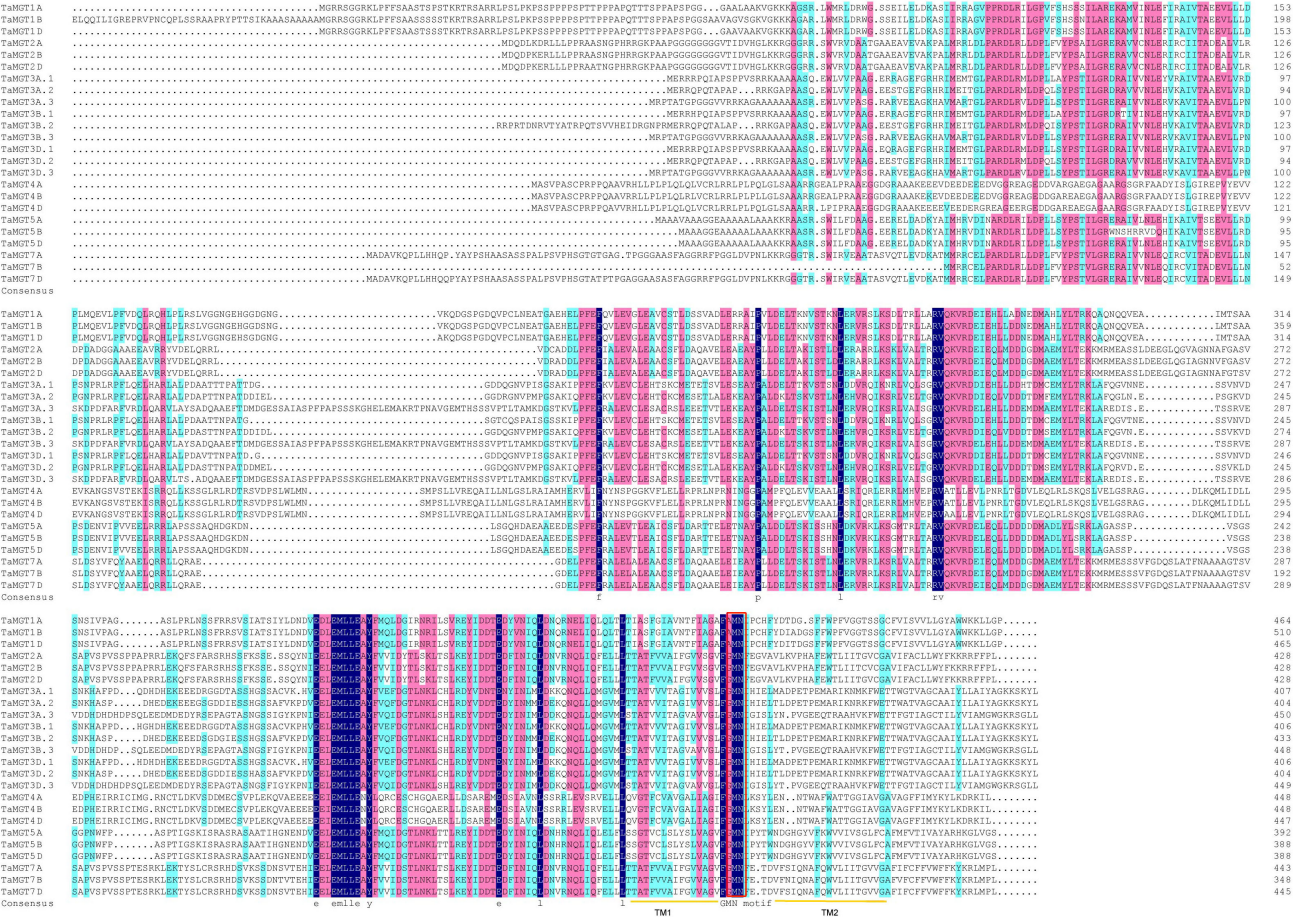
Multiple sequence alignment of TaMGTs. Multiple alignments were performed using DNAMAN software. TM domains are marked with orange lines. Conserved GMN motif is indicated by Red box.

Twenty-four putative *MGT* genes were identified in wheat. The candidate genes were named according to their chromosomal location (**Table 1**). The predicted molecular weights of TaMGTs were ranged from 39688.7D to 55522.41D. The protein isoelectric points were ranged from 4.74 to 7.26, of which TaMGT1A, TaMGT1B, and TaMGT1D had isoelectric points greater than 6, and more interestingly, they were triplet homologous genes. The protein instability index showed that only TaMGT5D was less than 40, which indicated that it was a stable protein. Analysis of the predicted hydrophobicity of the proteins showed that TaMGT7A and TaMGT7B are hydrophobic proteins. The results of subcellular localization analysis of these 24 proteins showed that 20 of them were widely localized in chloroplasts, and might also be localized in mitochondria and cytoplasm. TaMGT3A.3, TaMGT4A, TaMGT5B and TaMGT7B were only localized in the nucleus (**Table 1**). The TM domain analysis of TaMGT showed that similar to CorA/MRS2/MGT transport proteins in other species (including fungi), all 24 members contain two hypothetical TM domains at the C-terminus (**Supplementary Figure 1**) and cora-type domain. Multiple sequence alignment of TaMGTs showed that TM1 was more conserved than TM2. Moreover, the GMN tripeptide motif was found to be present in all members except for TaMGT1A, TaMGT1B, and TaMGT1D, which had the mutated motif AMN (alanine-methionine-aspartate) (**Figure 1**).

**Table 1:** TaMGTs protein features and prediction of subcellular location.

Gene name	Gene ID	Chr	Length	MW	TM	DM	pI	I. I	GRAVY	sub
*TaMGT1A*	*TraesCS1A03G0546100.1*	Chr1A:369306274-369308928	464	50775.05	2	cora	6.43	54.84	-0.167	Chl
*TaMGT1B*	*TraesCS1B03G0635400.1*	Chr1B:402375260-402377952	510	55522.41	2	cora	7.26	54.69	-0.163	Chl. Nuc
*TaMGT1D*	*TraesCS1D03G0526100.1*	Chr1D:297360401-297363124	465	50894.13	2	cora	6.23	53.96	-0.166	Chl.
*TaMGT2A*	*TraesCS2A03G0864500.1*	Chr2A:598758314-598762519	428	47298.31	2	cora	5.17	49.91	-0.048	Chl. Nuc
*TaMGT2B*	*TraesCS2B03G0965300.1*	Chr2B:544767428-544772321	428	47437.51	2	cora	5.24	49.93	-0.064	Chl. Nuc
*TaMGT2D*	*TraesCS2D03G0813900.1*	Chr2D:458190668-458194758	428	47407.42	2	cora	5.24	49.56	-0.082	Chl. Nuc
*TaMGT3A.1*	*TraesCS3A03G0899600.1*	Chr3A:631201551-631203919	408	45518.86	2	cora	5.33	45.03	-0.307	Chl. Nuc
*TaMGT3A.2*	*TraesCS3A03G0899700.1*	Chr3A:631206505-631210704	405	45290.65	2	cora	4.97	45.48	-0.351	Chl. Nuc.CM
*TaMGT3A.3*	*TraesCS3A03G0965700.1*	Chr3A:657786841-657791568	451	49332.86	2	cora	4.86	44.76	-0.22	Nuc
*TaMGT3B.1*	*TraesCS3B03G1023800.1*	Chr3B:665302551-665305027	407	45214.62	2	cora	5.58	43.68	-0.295	Chl. Nuc
*TaMGT3B.2*	*TraesCS3B03G1023900.1*	Chr3B:665306308-665311671	434	48638.26	2	cora	5.16	44.8	-0.41	Chl. Nuc .CM
*TaMGT3B.3*	*TraesCS3B03G1109400.1*	Chr3B:704250533-704254822	449	49127.73	2	cora	4.86	44.36	-0.191	Chl.
*TaMGT3D.1*	*TraesCS3D03G0826200.1*	Chr3D:487956862-487959377	407	45407.67	2	cora	5.26	42.69	-0.316	Chl.Nuc .CM .MN
*TaMGT3D.2*	*TraesCS3D03G0826300.1*	Chr3D:487960778-487965939	405	45395.67	2	cora	4.9	46.75	-0.358	Chl. Nuc .CM
*TaMGT3D.3*	*TraesCS3D03G0899300.1*	Chr3D:523593005-523597470	450	49246.81	2	cora	4.86	44.29	-0.208	Chl. Nuc
*TaMGT4A*	*TraesCS4A03G0733600.1*	Chr4A:590707040-590712507	448	49578.14	2	cora	5.43	57.92	-0.144	Nuc
*TaMGT4B*	*TraesCS4B03G0060700.1*	Chr4B:23432237-23437771	448	49639.31	2	cora	5.64	56.78	-0.151	Chl. Nuc
*TaMGT4D*	*TraesCS4D03G0040500.1*	Chr4D:11323563-11329187	447	49671.36	2	cora	5.71	57.78	-0.161	Chl. Nuc .CM
*TaMGT5A*	*TraesCS5A03G0916900.1*	Chr5A:582177688-582183695	392	43092.65	2	cora	4.85	40.17	-0.139	Chl. Nuc
*TaMGT5B*	*TraesCS5B03G0965000.1*	Chr5B:569002453-569007864	388	42905.33	2	cora	4.97	43.28	-0.218	Nuc
*TaMGT5D*	*TraesCS5D03G0876100.1*	Chr5D:463082622-463089037	388	42764.28	2	cora	4.85	39.98	-0.158	Chl. Nuc
*TaMGT7A*	*TraesCS7A03G1229000.1*	Chr7A:698716978-698721829	443	49161.28	2	cora	5.18	50.08	0.001	Chl
*TaMGT7B*	*TraesCS7B03G1115300.1*	Chr7B:689902980-689907578	348	39688.7	2	cora	4.74	50.65	0.082	Nuc
*TaMGT7D*	*TraesCS7D03G1169200.1*	Chr7D:604391025-604395163	445	49444.61	2	cora	5.18	50.82	-0.002	Chl

Chr, Chromosomal Location; Length, Amino acid length; MW, Molecular weight; pI, Isoelectric point; TM, Transmembranestructural domain; DM, cora domain; I.I, Instability index; GRAVY, Grand average of hydropathy; Sub, Subcellular localization; Nuc, Nucleus; Chl, Chloroplast; CM, Cytoplasm; MN, Mitochondrion.

### 3.2 Construction of phylogenetic tree, protein motif and gene structure analysis

**Figure 2:**
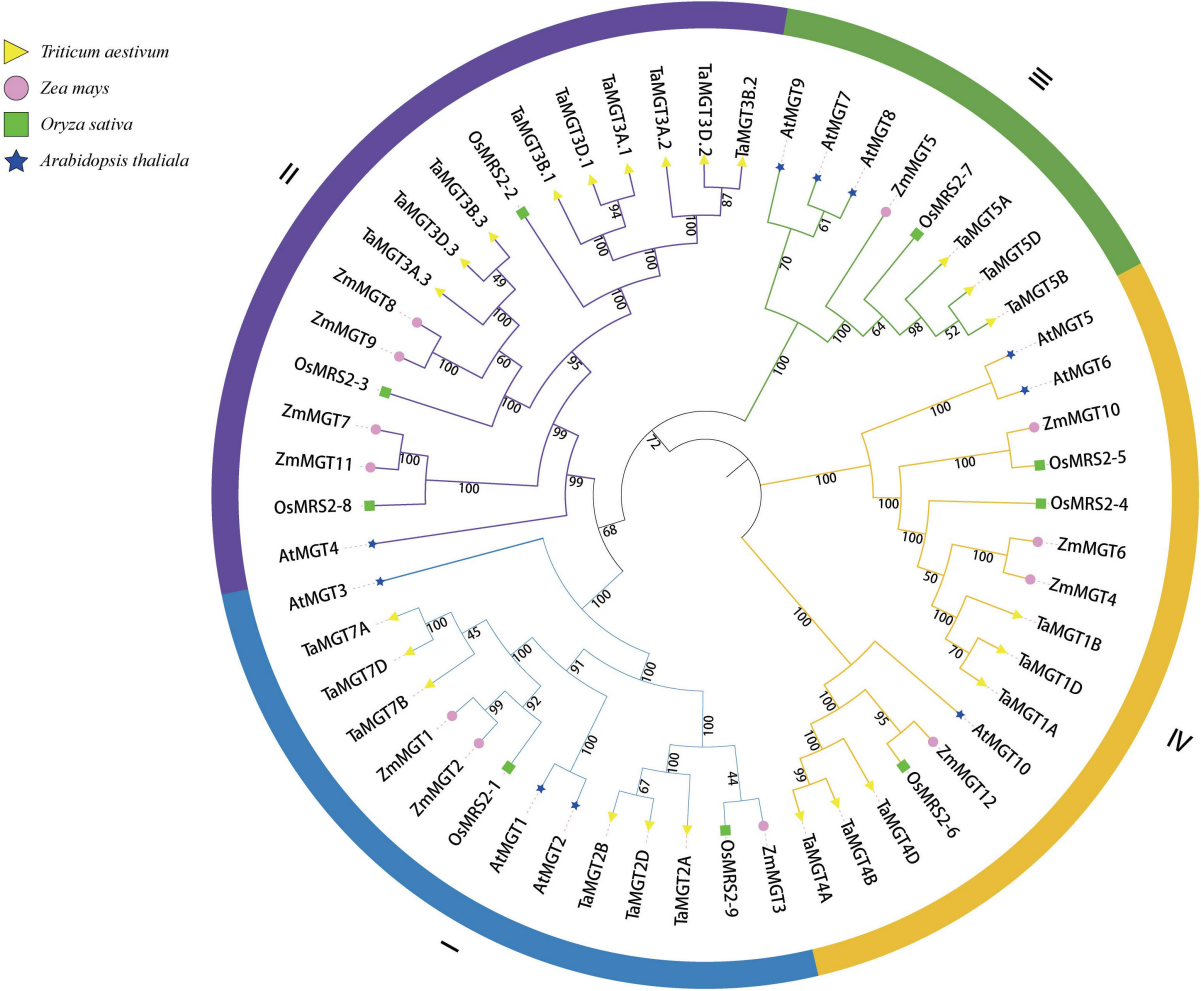
Phylogenetic analysis of Arabidopsis, rice, maize and wheat CorA/MRS2/MGT members. Phylogenetic trees were constructed by the neighbor-Joining method (NJ) using MEGA software for 24 TaMGT proteins, 11 Arabidopsis MRS2/MGT proteins, 9 rice proteins, and 12 maize proteins.

**Figure 3:**
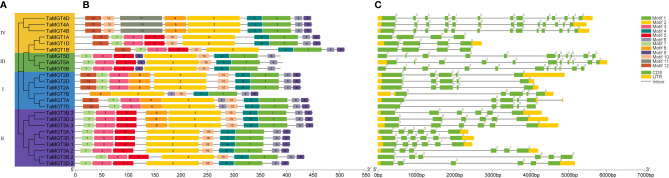
Phylogenetic relationships **(A)**, motif analysis **(B)** and gene structure **(C)** of TaMGT family members.

To better understand the evolutionary relationships of TaMGT with other species, phylogenetic trees were constructed for 10 Arabidopsis, 9 rice, 12 maize, and 24 wheat MGTs. As shown in **Figure 2**, all MGTs were divided into four groups, each group contained members from different plant species. Among them, the second group with the most proteins also has the most members of TaMGTs. The phylogenetic tree (**Figure 3**) showed that the 24 *TaMGTs* were also divided into four groups, which is consistent with the phylogenetic tree results of the above four plant species. Moreover, members from the same subgroup have similar motif types and structures. It is worth noting that *TaMGT1A*, *TaMGT1B*, *TaMGT1D* in Group IV lacked Motif 11, *TaMGT7B* in Group I lacked Motif 12, and *TaMGT7A*, *TaMGT7B*, *TaMGT7D* in Group I contained two Motif 6. The position of the motif varies from subgroup to subgroup, for example, Motif 9 is located at the 5’ end in group 3 and at the 3’ end in other subgroups. Motif 1 and Motif 6 were located in the functional structural domain of TaMGTs.

The results of the gene structure analysis (**Figure 3C**) showed that there are differences in exons/introns among the members in different subgroups. Almost all members of subgroup I and subgroup II contain 4-6 exons/introns and 2 non-coding regions, such as *TaMGT2B*, *TaMGT3B.3*, and *TaMGT3D.2*. Individual *TaMGT* genes do not have non-coding regions, such as *TaMGT7D*, *TaMG1A*, and *TaMG1B*. While in the fourth subgroup, *TaMGT4D*, *TaMGT4B*, and *TaMGT4A* contain 13 exons, 12 introns and 2 non-coding regions.

### 3.3 Chromosomal localization and gene duplication events

**Figure 4:**
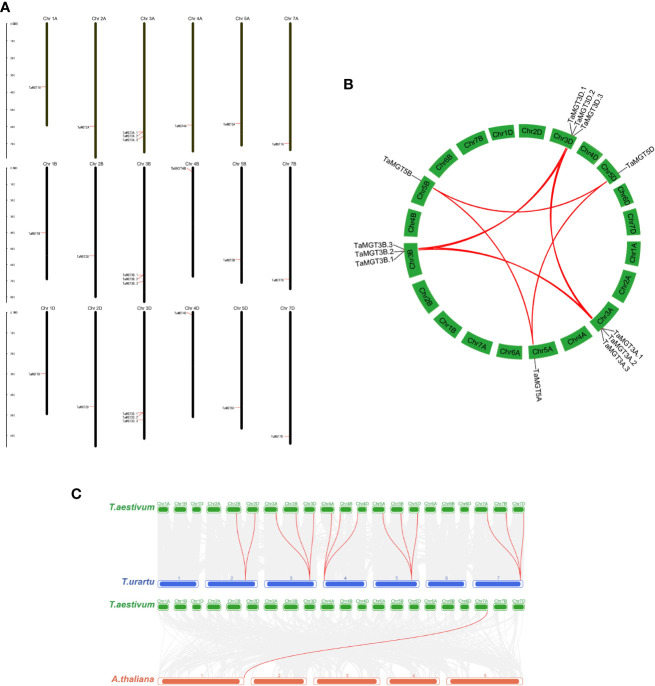
Chromosomal localization and gene duplication of TaMGTs genes. **(A)** Location of TaMGTs genes on chromosomes, with black representing chromosomes. **(B)** Gene duplication events in the wheat MGT gene family, where the curves represent gene duplication between chromosomes; the collinearity genes on chromosome 3A, 3B, 3C are highlighted with red lines. **(C)** Collinearity analysis of wheat with Arabidopsis thaliana and Triticum urartu. The gray line in the background indicates a collinear block in the genome of wheat and Arabidopsis thaliana and Triticum urartu, while the red line highlights the homologous MGT gene pair.

Based on the GFF3 annotation file, the chromosomal location of *TaMGT* genes were mapped using MapInspect software (**Figure 4A**). 24 *TaMGT* genes were distributed on 18 chromosomes. *TaMGT* genes were evenly distributed among the three subgenomes of wheat (each subgenome contained 8 genes). However, the distribution of the genes is uneven among the different chromosomes, with three *TaMGT* genes distributed on chromosome 3 and only one on the other chromosomes.

Gene duplication is one of the most important mechanisms by which organisms acquire new genes and create gene novelty (Song et al., 2019). Gene duplication consists of both tandem duplication and segmental duplication (Vision et al., 2000). In order to obtain the amplification mechanism of the *TaMGT* genes, the collinearity among *TaMGT* genes was analyzed by MCScanX software (**Figure 4B**). The results showed that there were twelve pairs of segmental duplication events among *TaMGT* genes.And most of the duplicated *TaMGT* genes were located on different chromosomes, and no tandem duplication relationships were found among *TaMGT* members on the same chromosome. These results suggest that the *TaMGT* genes may have undergone segmental replications during evolution.

To further analyze the evolutionary and homologous relationships of the MGT family in wheat, collinearity analysis of wheat with *Arabidopsis thaliana* and *Triticum urartu* were constructed. The results (**Figure 4C**) showed that there were 14 *MGT* homologous gene pairs in wheat and its close relative *Triticum urartu*, while there were only one *MGT* homologous gene pair in wheat and dicotyledonous *Arabidopsis*, which might be due to the short differentiation distance between wheat and its close relatives, with fewer events such as gene loss, insertion and transposition.

### 3.4 Analysis of Cis-acting elements and transcriptome expression analysis

**Figure 5:**
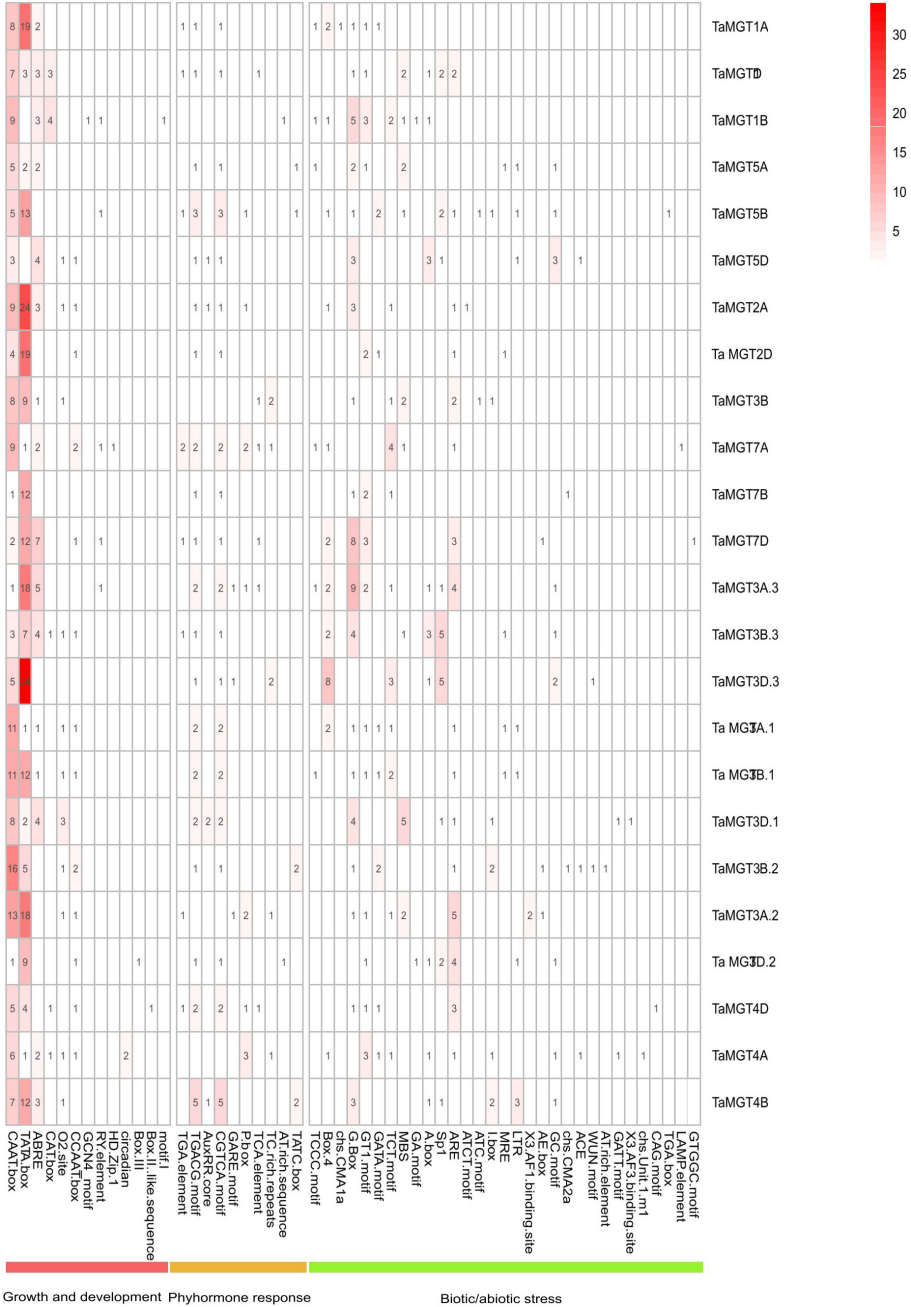
Cis-acting elements analysis of TaMGT genes.

Cis-acting regulatory elements were bound by appropriate transcription factors to control gene transcription (Liu et al., 2013). In this experiment, we identified and summarized the cis-acting elements in the promoter region of the *TaMGT* gene family related to growth and development, biotic stress and phytohormones (**Figure 5** and **Supplemental Table 2**), and found that there were differences in the number and types of elements contained in the promoters of the *TaMGT* family. *TaMGT3D.3* promoter contained 69 cis-acting elements, while *TaMGT7B* just contained 20. Among the cis-elements associated with growth and development, the TATA-box was the most. Among the hormone-responsive cis-elements, TGACG-motif and CGTCA-motif were involved in the regulation of the methyl jasmonate response. Among the biotic and abiotic stress-related cis-elements, WUN-motif is a wound responsive element, MYB is a drought-inducibility element, ARE and GC-motif are an anaerobic induction element, and LTR is a low-temperature response element (Bang et al., 2013; Banerjee et al., 2013; Mittal et al., 2009; Kovalchuk et al., 2013; Li et al., 2022). These results suggest that this gene family may also play an important function in the response to adversity stresses.

**Figure 6:**
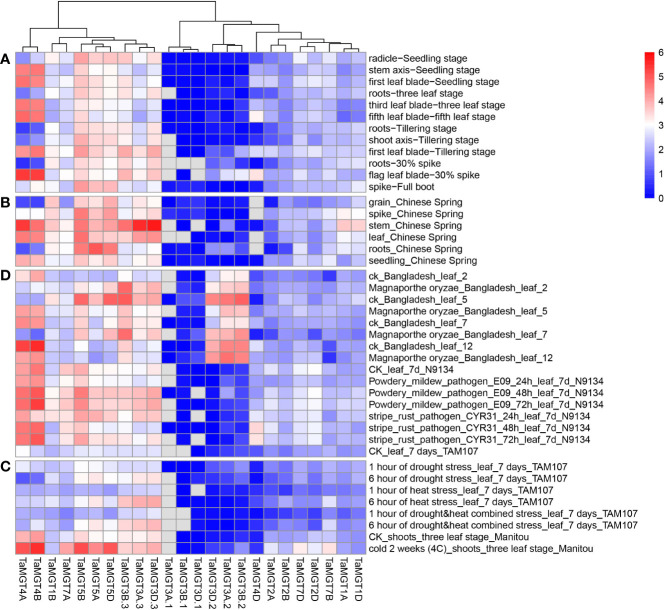
**(A)** different growth and developmental stages expression analysis **(B)** different tissues expression analysis **(C)** abiotic stresses expression analysis **(D)** biotic stresses expression analysis.

We used available wheat RNA-seq data (Supplemental information: **Tables S3, S4**) to analyze the expression levels of the *TaMGT* gene at different growth and developmental stages of wheat, and the results were shown in **Figure 6**. These *TaMGTs* were expressed in grains, headings, stems, leaves, roots, and seeds. According to the clustering results of their expression levels, these *TaMGTs* can be divided into four groups, in which the genes of Groups 2 and 3 were expressed or lowly expressed in all tissues, while the expression levels of genes in Group 4 were much higher. For example, *TaMGT5D*, *TaMGT5A*, and *TaMGT5B* genes were highly expressed in all five tissues; *TaMGT3D.3* and *TaMGT3A.3* were abundantly expressed in stems, but not expressed or lowly expressed in other tissues, indicating that *TaMGT3D.3* and *TaMGT3A.3* may play important roles in stem development. In addition, the expression levels of these genes in the seeds also varied, with ten genes expressed in small amounts and the remaining genes hardly expressed. These results suggest that the differential expression of *TaMGTs* may play important but distinct roles in different tissues.

In addition to detecting the expression levels of each *TaMGTs* in different tissues, we also analyzed their expressions at different developmental stages. Based on the clustering results of their expression levels, *TaMGT4A* and *TaMGT4B* were highly expressed at the stem axis-seedling stage, the first leaf-seedling stage, the third leaf-three-leaf stage, the first leaf tillering stage, the root-three-leaf stage, and the flag leaf-30% Ear stage. While almost no expression was found in other periods. These two genes may be mainly involved in the regulation of these six tissue developmental stages in wheat.

Next, we analyzed the expression differences of *TaMGTs* under biotic and abiotic stresses. As shown in **Figure 6C**, all *TaMGTs* genes were not significantly induced under drought stress. In drought stress treatment, the expression level of *TaMGT5A*, *TaMGT3A.3*, and *TaMGT3D.3* genes gradually increased with the increaseing of treatment time, while the expression of *TaMGT4A* and *TaMGT4B* genes gradually decreased. It can be seen that the genes in the 4 groups were affected to a certain extent by the increase of processing time. The expression levels of *TaMGT3D.3*, *TaMGT3A.3*, and *TaMGT3B.3* increased significantly with the prolongation of drought stress, heat stress, and drought and heat stress, indicating that these genes may be related to the degree of abiotic stress in plants.

After cold treatment, the expression of *TaMGT5D*, *TaMGT5B*, *TaMGT4A*, and *TaMGT4B* genes was significantly upregulated, which indicated that these genes might play positive roles against cold stress. Among them, *TaMGT4A* and *TaMGT4B* were barely expressed in drought stress, thermal stress, and drought and heat stress, and it is speculated that these two genes may be related to the cold resistance of plants.

In addition, under biotic stress, most *TaMGTs* had different gene expression patterns after *Magnaporthe oryzae* infection, indicating that these *TaMGTs* responded differently to rice blast (**Figure 6D**). The genes in Group 1 and 3 were unexpressed or low-expressed in all tissues, but those in Group 2 and 4 were expressed at much higher levels and responded significantly to different treatments. After the fifth day of infection, the gene expression level of *TaMGT3B.2*, *TaMGT3A.2*, *TaMGT3D.2*, *TaMGT3B.3*, *TaMGT5D*, and *TaMGT5B* were significantly down-regulated. However, with the increase of infection time, the expression levels of *TaMGT4A* and *TaMBT4B* were gradually increased. Therefore, we speculate that *TaMGT4A* and *TaMGT4B* may be involved in the response to *Magnaporthe oryzae* infection.

When plants were inoculated with powdery mildew and stripe rust, the expression patterns of *TaMGT* genes were similar in response to these two diseases. As the time of infection increases, the expression level of some genes were increased significantly. Among them, *TaMGT4A* and *TaMGT4B* had significant responses to powdery mildew and stripe rust. Combined with the previous analysis, it is speculated that these two genes are mainly related to the regulation of plant disease resistance.

### 3.5 Response characteristics of *TaMGT* family genes to Mg^2+^ deficiency, AL stress, and ABA treatment

We analyzed the expression of *TaMGT* gene in roots and shoots under Mg^2+^ deficiency conditions using qRT-PCR. The results showed that under the condition of Mg^2+^ deficiency, the expression level of *TaMGT1A* was strongly upregulated and peaked at 48h in the leaf and peaked at 6h in the root, and *TaMGT5B* peaked at 24h in leaf. Compared with the untreated group, *TaMGT3B.3*, *TaMGT4B* and *TaMGT7A* showed a downward trend in leaves and roots. These results indicated that the *TaMGT* genes responded differently to magnesium deficiency.

**Figure 7:**
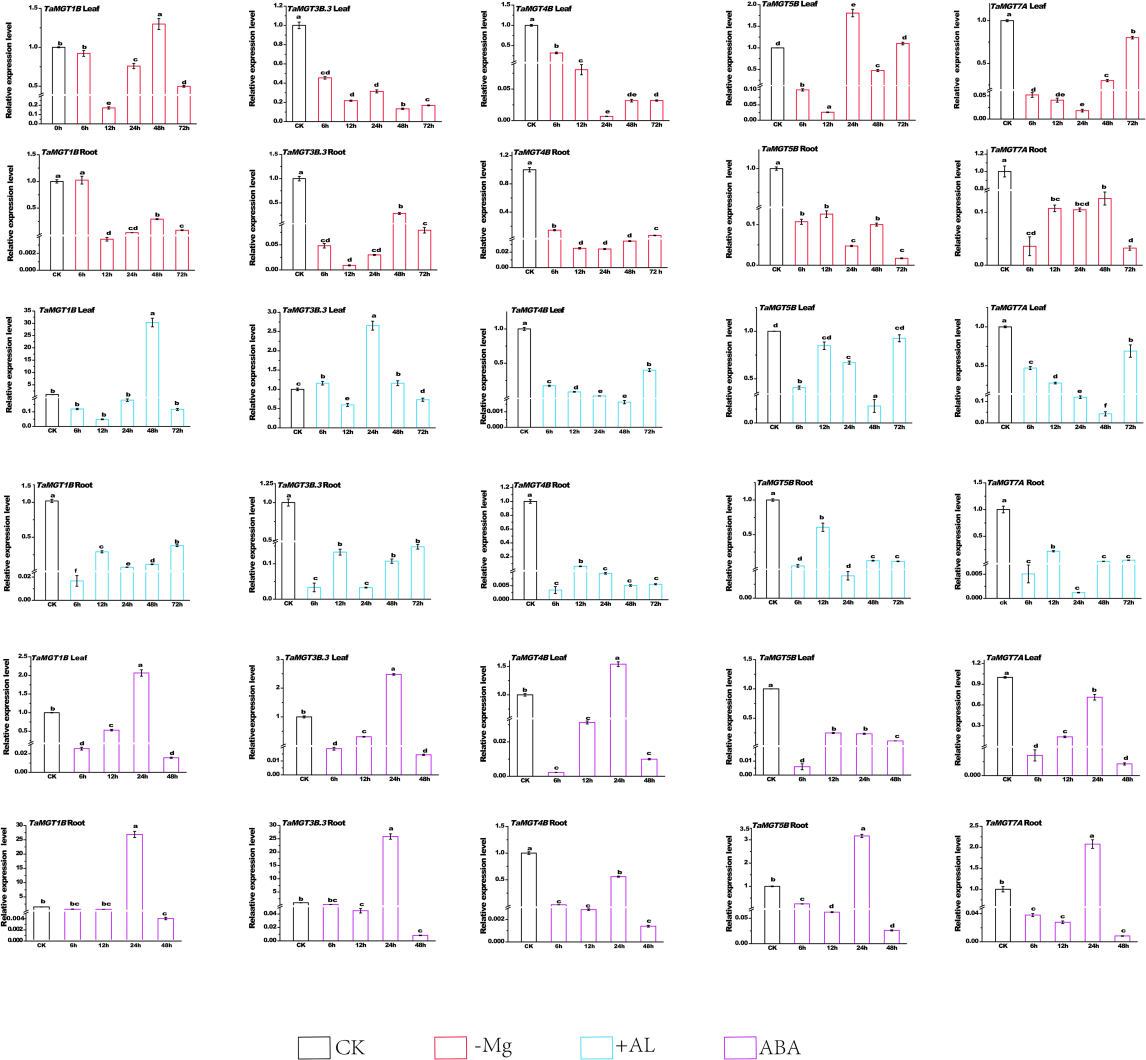
Expression patterns of five *TaMGT* genes under three different treatments. Three groups of treatments included 60uM aluminum chloride, 100 mmol/L ABA treatment, and magnesium deficiency treatment. The Y-axis represents the relative expression level, and the X-axis represents the time point of treatment. Significant differences at P<0.05 are indicated by letters. The expression levels of *TaMGT* genes were mapped using Origin software.

Compared with the control group, the gene expression levels of *TaMGT1B* and *TaMGT3B* reached the maximum at 24h and 48h respectively (**Figure 7**) after the plant leaves were exposed to aluminum stress, which was about 10 and 2.75 times higher than those of the control group, respectively, indicating that these two genes may be involved in the regulation of aluminium stress. In addition, the gene expression levels of *TaMGT4B*, *TaMGT5B*, and *TaMGT7A* were significantly decreased during each period, and the expression levels of *TaMGT4B* and *TaMGT7A* were gradually down-regulated before 48h, which may be caused by the stress response of plants.

Compared with the control group, the expression of all genes was inhibited after the roots of the plant were subjected to aluminum stress. The expression levels of *TaMGT5B* and *TaMGT7A* showed a significantly downward trend and began to level-off after 48h. The expression levels of *TaMGT3B.3* and *TaMGT7A* showed the same trend at 6h, 12h, 24h and 48h, with *TaMGT7A* levelling off after 72h and *TaMGT3B.3* having a continued up-regulation trend with increasing treatment time. Gene expression in roots and leaves indicated that gene expression was inhibited under Al stress.

The expression patterns of *TaMGTs* showed (**Figure 7**) that after 24 h of abscisic acid treatment, *TaMGT5B* and *TaMGT7A* were down-regulated in the leaves and *TaMGT4B* was down-regulated in the roots. The expression levels of other *TaMGT* genes peaked in both leaves and roots 24 h after abscisic acid treatment. The expression levels of most *TaMGT* genes showed a tendency to descend and then rise under abscisic acid treatment. The above results show that the *TaMGT* family gene has a different role in wheat response to abscisic acid treatment.

## 4 Discussion

Although the magnesium transporter protein family genes have been extensively studied in a large number of plant species(Schock et al., 2000; Saito et al., 2013; Zhao et al., 2018; Liu et al., 2019; Heidari et al, 2022), no reports in wheat was published. In this study, 24 *TaMGT* genes were identified for the first time in the wheat genomeusing bioinformatics methods.

The results of gene structure and motif analysis showed that the deletion of Motif 11 in *TaMGT1A*, *TaMGT1B*, *TaMGT1D* and the deletion of Motif 12 in *TaMGT7B* may be caused by gene recombination (Melamed-Bessudo et al., 2016), while further experiments are needed to prove it. In addition, members of the MGT family had variations in the number of exons. The exon number can increase the diversity of gene-coding proteins by influencing post-transcriptional processes, such as alternative splicing (Koralewski and Krutovsky, 2011). Those with fewer exons can activate rapidly in response to stress, and these genes play a stronger role in adapting to adverse environmental conditions (Jeffares et al., 2008; Jiang et al., 2022).There were segmental duplication relationships between genes involved in gene duplication in *TaMGT* gene family, with no signs of tandem duplication relationships (**Figure 4B**), which is consistent with the previous report of interrelationships of genes in the *MGT* gene family in other species (Yan et al., 2019). Therefore, it is speculated that the expansion of MGT family caused by segmental duplication plays an important role in the adaptation of wheat to external environmental changes.

Cis-regulatory elements in promoter regions play important roles in the regulation of gene expression (Hernandez-Garcia and Finer, 2014). *MGT* genes have a high potential to respond to stresses, including hormones, growth and development, cis-related elements of biological stress and abiotic stress. It shows that this family plays a major role in the growth and development of wheat and adapts to the external environments (such as changes in light signal, hormones and stress), and also indicates that there are multiple factors regulating the expression of *TaMGT* gene. Among them, the TaMGT promoter contained the growth and development response elements was the largest, indicating that *TaMGT* gene may affect the growth and development of wheat.

Differential analysis of the wheat transcriptome revealed significant differences in the *TaMGT* family of genes under diverse environmental stresses. *TaMGT4A* and *TaMGT4B* maintained high expression levels under different stress conditions. However, *TaMGT3B.2*, *TaMGT3A.2*, and *TaMGT3D.2* were only expressed under the stress of rice blast. There are few studies on magnesium transporters in response to disease stress. Watermelon *MSR2* gene were upregulated in response to mosaic virus stress(Heidari et al, 2022). *Arabidopsis MGT* gene, *AT2G21120*, which was recognized using a forward genetic screen, was involved in response to viral infection (Guo et al., 2018). Further studies of the functions of these genes are needed to elucidate the specific mechanisms involved in these phenomena.

Magnesium deficiency has been reported to have an effect on *MGT* gene expression (Li et al., 2016; Tong et al., 2020), so we analyzed the expression levels of the *MGT* gene in wheat under the condition of magnesium deficiency by qRT-PCR. The results showed that the expression levels of five *TaMGT* genes were significantly different in wheat leaves and roots. Studies have shown that *AtMGT6* and *ZmMGT10* are essential for maintaining Mg homeostasis and are highly expressed in roots under Mg deficiency (Mao et al., 2014; Li et al., 2017; Tang and Luan, 2017). Phylogenetic analysis showed that *TaMGT1B*, a homologue of *AtMGT6* and *ZmMGT10*, was significantly up-regulated in Mg-deficient roots and leaves. *AtMGT6* mediates Mg2+ uptake in roots and is required for plant adaptation to a low-Mg^2+^ environment (Mao et al., 2014). Whether *TaMGT1B* has the same function needs further experiments. *AtMGT7* is involved in Mg uptake and can maintain normal Arabidopsis growth and development under low Mg or Mg-deficient conditions (Mao et al., 2008). *TaMGT5B*, a homolog of *AtMGT7* and *OsMRS2-7*, was highly upregulated in leaves. It has been shown that branch A of *OSMRS2-6* and *AtMRS2-11* has been defined as chloroplast transporter group (Saito et al., 2013), *TaMGT4A*, *TaMGT4B*, *TaMGT4D*, *OSMRS2-6*, and *AtMRS2-11* were belonged to the same branch (**Figure 2**), and they may be related to chloroplast metabolism.

Studies have demonstrated that magnesium transporters can alleviate the toxic of Al in the absence of magnesium. Overexpression of Mg transporters (ALR1 or ALR2) in *Saccharomyces cerevisiae* confers Al tolerance in yeast (MacDiarmid and Gardner, 1998). The *OsMGT1* gene, a plasma membrane-localized transporter of Mg in rice, confers Al tolerance by increasing Mg uptake into cells (Chen et al., 2012). Most of the *MGT* genes in Arabidopsis were not up-regulated by Al treatment (Hoekenga et al., 2003; Sawaki et al., 2009; Zhao et al., 2009). In this study, it was found that most of the *MGT* genes in wheat were also not up-regulated by Al. This is consistent with related reports that rice is highly tolerant to Al toxicity (Chen et al., 2012).The transgenic lines for *AtMGT1* in *Nicotiana benthamiana* showed a reduction in Al toxicity (Deng et al., 2006). It seems that increasing MGT activity plays an important role in reducing the negative effects of some elements and ions. In the sugarcane MGT family, different genes were expressed differently under different ABA treatment times. The five *TaMGT* genes (*TaMGT1B*, *TaMGT3B.3*, *TaMGT4B*, *TaMGT5B* and *TaMGT7A*) in wheat have the same expression pattern at 12-48h under abscisic acid treatment, which may indicate that these five genes play some function in response to ABA. However, the exact functions performed need to be further verified.

In summary, this study identified 24 MGT-type magnesium transporter proteins and provided a detailed analysis of their classification, protein structure, evolutionary relationships, gene structure and localization. Changes in the CorA GMN motif in *Salmonella typhimurium* have been used as zinc transporter proteins, which may mediate the entry and exit of Zn but are unable to transport Mg^2+^ (Worlock and Smith, 2002). Given this situation, it remains to be demonstrated whether mutation of the GMN motif to AMN in wheat has a function in transporting Mg. Transcriptomic analysis and qRT-PCR analysis showed that *TaMGTs* play an important role in responding to diverse abiotic stresses. *TaMGT* genes are differentially expressed under Mg-deficient conditions, aluminium stress and abscisic acid treatment. Among them, *TaMGT1B* may play a prominent function in maintaining Mg homeostasis, Mg uptake and participation in hormone response in plants. The results of this study provide a theoretical basis for an in-depth study of the function of the *TaMGT* gene family in wheat.

## 5 Conclusions

In this study, the classification, protein structure, evolutionary relationship, gene structure and localization of 24 *TaMGT* genes in wheat were analyzed in detail. The quantitative PCR results suggested that *TaMGT* plays a role in maintaining magnesium homeostasis, magnesium absorption and hormone response in wheat, as well as in response to biological and non-organic important role in biotic stress. The results provide a theoretical basis for further research on the function of *TaMGT* gene family in wheat.

## Data availability statement

The datasets presented in this study can be found in online repositories. The names of the repository/repositories and accession number(s) can be found in the article/**Supplementary material**.

## Author contributions

ZL, DM, and WC conceived the study and worked on the approval of the manuscript. YT, XY, and HL performed the experiments and wrote the first draft. WC, DM, and ZL revised the manuscript. YS contributed to data analysis. All authors contributed to the article and approved the submitted version.
