# Supplementary material for: Uncovering the role of wheat magnesium transporter family genes in abiotic responses
Source: Front Plant Sci. 2023 Feb 9;14:1078299. doi: 10.3389/fpls.2023.1078299 (PMC9948656; doi:10.3389/fpls.2023.1078299)
Supplement: Supplementary file 1 [file DataSheet_1.docx]

Supplementary Material

# Supplementary Figures and Tables

## Supplementary Figures


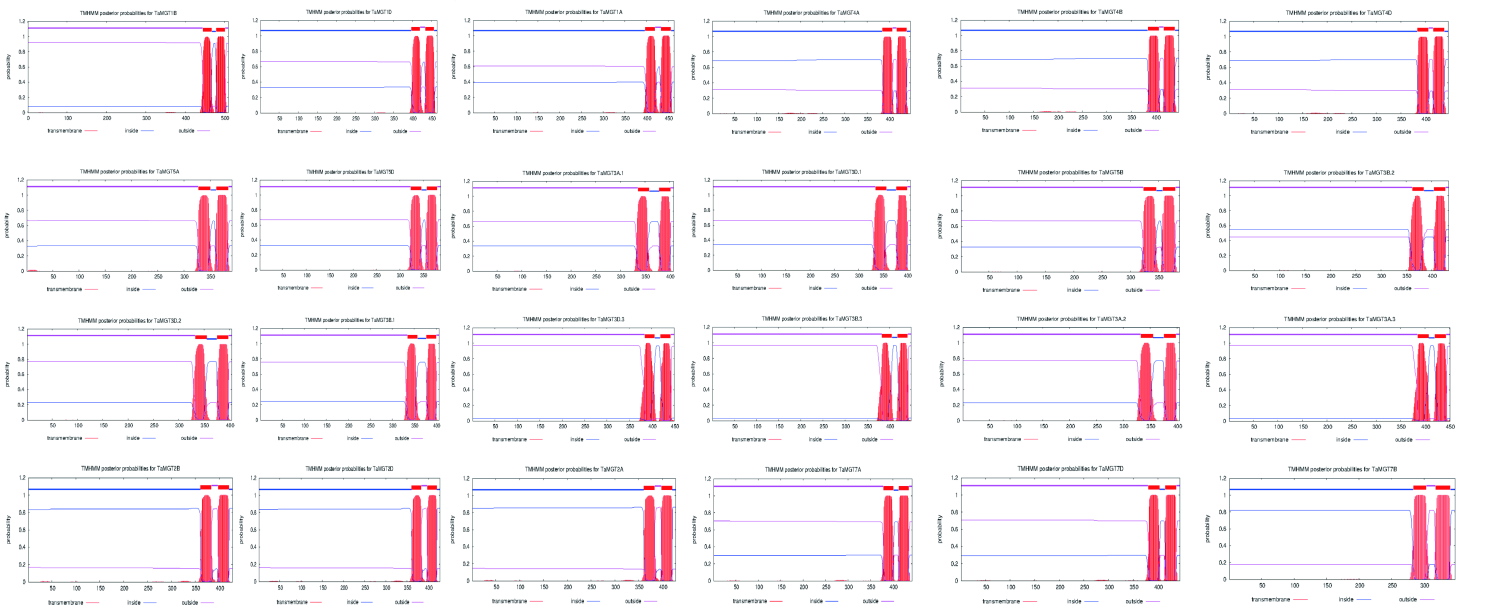


**Supplementary Figure 1.** Predicted TM regions of wheat MRS2/MGT proteins Predicted TM region of wheat MRS2/MGT proteins using TMH MM2 (http://www.cbs.dtu.dk/services/TMHMM 2.0/). Red peaks i ndicate predicted transmembrane.

## Supplementary Table

**Supplementary Table 1.** PCR primers used for Real-Time PCR

| Gene name | Forward (5’-3’) | Reverse (5’-3’) |
| --- | --- | --- |
| *TaMGT1B* | GGAGAAGGAGGTGGATGAGG | TGTTCACCATTCCCACCCAC |
| *TaMGT3B.3* | ATGACTCATAGCAGCGGTGCCC | AGATGCTCAAGCTCATCCCTCACC |
| *TaMGT4B* | GATACAAGAAGCGTTGACCCGTCAC | TGAAAAGGCATTGCAGGCCCAC |
| *TaMGT5B* | CCCACAGAATCACGGAAGTTG | CATAAGCGACAATCGTGACAAAC |
| *TaMGT7A* | CGACAGCACTCTTAACAAGCTGACC | AGAAGCAGAAGATGAAAGCGCCAAC |
| *Ta2291* | GCTCTCCAACAACATTGCCAAC | GCTTCTGCCTGTCACATACGC |

**Supplementary Table 2.** Numbers of stress-related cis-elements in upstream 1.5 kb regions of *TaMGT* genes

|  | TaMGT1A | TaMGT1D | TaMGT1B | TaMGT5A | TaMGT5B | TaMGT5D | TaMGT2A | TaMGT2D | TaMGT2B | TaMGT7A | TaMGT7B | TaMGT7D | TaMGT3A.3 | TaMGT3B.3 | TaMGT3D.3 | TaMGT3A.1 | TaMGT3B.1 | TaMGT3D.1 | TaMGT3B.2 | TaMGT3A.2 | TaMGT3D.2 | TaMGT4D | TaMGT4A | TaMGT4B |
| --- | --- | --- | --- | --- | --- | --- | --- | --- | --- | --- | --- | --- | --- | --- | --- | --- | --- | --- | --- | --- | --- | --- | --- | --- |
| CAAT-box | 8 | 7 | 9 | 5 | 5 | 3 | 9 | 4 | 8 | 9 | 1 | 2 | 1 | 3 | 5 | 11 | 11 | 8 | 16 | 13 | 1 | 5 | 6 | 7 |
| TATA-box | 19 | 3 | 0 | 2 | 13 | 0 | 24 | 19 | 9 | 1 | 12 | 12 | 18 | 7 | 34 | 1 | 12 | 2 | 5 | 18 | 9 | 4 | 1 | 12 |
| ABRE | 2 | 3 | 3 | 2 | 0 | 4 | 3 | 0 | 1 | 2 | 0 | 7 | 5 | 4 | 0 | 1 | 1 | 4 | 0 | 0 | 0 | 0 | 2 | 3 |
| CAT-box | 0 | 3 | 4 | 0 | 0 | 0 | 0 | 0 | 0 | 0 | 0 | 0 | 0 | 1 | 0 | 0 | 0 | 0 | 0 | 0 | 0 | 1 | 1 | 0 |
| O2-site | 0 | 0 | 0 | 0 | 0 | 1 | 1 | 0 | 1 | 0 | 0 | 0 | 0 | 1 | 0 | 1 | 1 | 3 | 1 | 1 | 0 | 0 | 1 | 1 |
| CCAAT-box | 0 | 0 | 0 | 0 | 0 | 1 | 1 | 1 | 0 | 2 | 0 | 1 | 0 | 1 | 0 | 1 | 1 | 0 | 2 | 1 | 1 | 1 | 1 | 0 |
| GCN4_motif | 0 | 0 | 1 | 0 | 0 | 0 | 0 | 0 | 0 | 0 | 0 | 0 | 0 | 0 | 0 | 0 | 0 | 0 | 0 | 0 | 0 | 0 | 0 | 0 |
| RY-element | 0 | 0 | 1 | 0 | 1 | 0 | 0 | 0 | 0 | 1 | 0 | 1 | 1 | 0 | 0 | 0 | 0 | 0 | 0 | 0 | 0 | 0 | 0 | 0 |
| HD-Zip 1 | 0 | 0 | 0 | 0 | 0 | 0 | 0 | 0 | 0 | 1 | 0 | 0 | 0 | 0 | 0 | 0 | 0 | 0 | 0 | 0 | 0 | 0 | 0 | 0 |
| circadian | 0 | 0 | 0 | 0 | 0 | 0 | 0 | 0 | 0 | 0 | 0 | 0 | 0 | 0 | 0 | 0 | 0 | 0 | 0 | 0 | 0 | 0 | 2 | 0 |
| Box III | 0 | 0 | 0 | 0 | 0 | 0 | 0 | 0 | 0 | 0 | 0 | 0 | 0 | 0 | 0 | 0 | 0 | 0 | 0 | 0 | 1 | 0 | 0 | 0 |
| Box II -like sequence | 0 | 0 | 0 | 0 | 0 | 0 | 0 | 0 | 0 | 0 | 0 | 0 | 0 | 0 | 0 | 0 | 0 | 0 | 0 | 0 | 0 | 1 | 0 | 0 |
| motif I | 0 | 0 | 1 | 0 | 0 | 0 | 0 | 0 | 0 | 0 | 0 | 0 | 0 | 0 | 0 | 0 | 0 | 0 | 0 | 0 | 0 | 0 | 0 | 0 |
| TGA-element | 1 | 1 | 0 | 0 | 1 | 0 | 0 | 0 | 0 | 2 | 0 | 1 | 0 | 1 | 0 | 0 | 0 | 0 | 0 | 1 | 0 | 1 | 0 | 0 |
| TGACG-motif | 1 | 1 | 0 | 1 | 3 | 1 | 1 | 1 | 0 | 2 | 1 | 1 | 2 | 1 | 1 | 2 | 2 | 2 | 1 | 0 | 1 | 2 | 0 | 5 |
| AuxRR-core | 0 | 0 | 0 | 0 | 0 | 1 | 1 | 0 | 0 | 0 | 0 | 0 | 0 | 0 | 0 | 0 | 0 | 2 | 0 | 0 | 0 | 0 | 0 | 1 |
| CGTCA-motif | 1 | 1 | 0 | 1 | 3 | 1 | 1 | 1 | 0 | 2 | 1 | 1 | 2 | 1 | 1 | 2 | 2 | 2 | 1 | 0 | 1 | 2 | 0 | 5 |
| GARE-motif | 0 | 0 | 0 | 0 | 0 | 0 | 0 | 0 | 0 | 0 | 0 | 0 | 1 | 0 | 1 | 0 | 0 | 0 | 0 | 1 | 0 | 0 | 0 | 0 |
| P-box | 0 | 0 | 0 | 0 | 1 | 0 | 1 | 0 | 0 | 2 | 0 | 0 | 1 | 0 | 0 | 0 | 0 | 0 | 0 | 2 | 0 | 1 | 3 | 0 |
| TCA-element | 0 | 1 | 0 | 0 | 0 | 0 | 0 | 0 | 1 | 1 | 0 | 1 | 1 | 0 | 0 | 0 | 0 | 0 | 0 | 0 | 0 | 1 | 0 | 0 |
| TC-rich repeats | 0 | 0 | 0 | 0 | 0 | 0 | 0 | 0 | 2 | 1 | 0 | 0 | 0 | 0 | 2 | 0 | 0 | 0 | 0 | 1 | 0 | 0 | 1 | 0 |
| AT-rich sequence | 0 | 0 | 1 | 0 | 0 | 0 | 0 | 0 | 0 | 0 | 0 | 0 | 0 | 0 | 0 | 0 | 0 | 0 | 0 | 0 | 1 | 0 | 0 | 0 |
| TATC-box | 0 | 0 | 0 | 1 | 1 | 0 | 0 | 0 | 0 | 0 | 0 | 0 | 0 | 0 | 0 | 0 | 0 | 0 | 2 | 0 | 0 | 0 | 0 | 2 |
| TCCC-motif | 1 | 0 | 1 | 1 | 0 | 0 | 0 | 0 | 0 | 1 | 0 | 0 | 1 | 0 | 0 | 0 | 1 | 0 | 0 | 0 | 0 | 0 | 0 | 0 |
| Box 4 | 2 | 0 | 1 | 0 | 1 | 0 | 1 | 0 | 0 | 1 | 0 | 2 | 2 | 2 | 8 | 2 | 0 | 0 | 0 | 0 | 0 | 0 | 1 | 0 |
| chs-CMA1a | 1 | 0 | 0 | 0 | 0 | 0 | 0 | 0 | 0 | 0 | 0 | 0 | 0 | 0 | 0 | 0 | 0 | 0 | 0 | 0 | 0 | 0 | 0 | 0 |
| G-Box | 1 | 1 | 5 | 2 | 1 | 3 | 3 | 0 | 1 | 0 | 1 | 8 | 9 | 4 | 0 | 1 | 1 | 4 | 1 | 1 | 0 | 1 | 0 | 3 |
| GT1-motif | 1 | 1 | 3 | 1 | 0 | 0 | 0 | 2 | 0 | 0 | 2 | 3 | 2 | 0 | 0 | 1 | 1 | 0 | 0 | 1 | 1 | 1 | 3 | 0 |
| GATA-motif | 1 | 0 | 0 | 0 | 2 | 0 | 0 | 1 | 0 | 0 | 0 | 0 | 0 | 0 | 0 | 1 | 1 | 0 | 2 | 0 | 0 | 1 | 1 | 0 |
| TCT-motif | 0 | 0 | 2 | 0 | 0 | 0 | 1 | 0 | 1 | 4 | 1 | 0 | 1 | 0 | 3 | 1 | 2 | 0 | 0 | 1 | 0 | 0 | 1 | 0 |
| MBS | 0 | 2 | 1 | 2 | 1 | 0 | 0 | 0 | 2 | 1 | 0 | 0 | 0 | 1 | 0 | 0 | 0 | 5 | 0 | 2 | 0 | 0 | 0 | 0 |
| GA-motif | 0 | 0 | 1 | 0 | 0 | 0 | 0 | 0 | 0 | 0 | 0 | 0 | 0 | 0 | 0 | 0 | 0 | 0 | 0 | 0 | 1 | 0 | 0 | 0 |
| A-box | 0 | 1 | 1 | 0 | 0 | 3 | 0 | 0 | 0 | 0 | 0 | 0 | 1 | 3 | 1 | 0 | 0 | 0 | 0 | 0 | 1 | 0 | 1 | 1 |
| Sp1 | 0 | 2 | 0 | 0 | 2 | 1 | 0 | 0 | 0 | 0 | 0 | 0 | 1 | 5 | 5 | 0 | 0 | 1 | 0 | 0 | 2 | 0 | 0 | 1 |
| ARE | 0 | 2 | 0 | 0 | 1 | 0 | 1 | 1 | 2 | 1 | 0 | 3 | 4 | 0 | 0 | 1 | 1 | 1 | 1 | 5 | 4 | 3 | 1 | 0 |
| ATCT-motif | 0 | 0 | 0 | 0 | 0 | 0 | 1 | 0 | 0 | 0 | 0 | 0 | 0 | 0 | 0 | 0 | 0 | 0 | 0 | 0 | 0 | 0 | 0 | 0 |
| ATC-motif | 0 | 0 | 0 | 0 | 1 | 0 | 0 | 0 | 1 | 0 | 0 | 0 | 0 | 0 | 0 | 0 | 0 | 0 | 0 | 0 | 0 | 0 | 0 | 0 |
| I-box | 0 | 0 | 0 | 0 | 1 | 0 | 0 | 0 | 1 | 0 | 0 | 0 | 0 | 0 | 0 | 0 | 0 | 1 | 2 | 0 | 0 | 0 | 1 | 2 |
| MRE | 0 | 0 | 0 | 1 | 0 | 0 | 0 | 1 | 0 | 0 | 0 | 0 | 0 | 1 | 0 | 1 | 1 | 0 | 0 | 0 | 0 | 0 | 0 | 0 |
| LTR | 0 | 0 | 0 | 1 | 1 | 1 | 0 | 0 | 0 | 0 | 0 | 0 | 0 | 0 | 0 | 1 | 1 | 0 | 0 | 0 | 1 | 0 | 0 | 3 |
| 3-AF1 binding site | 0 | 0 | 0 | 0 | 0 | 0 | 0 | 0 | 0 | 0 | 0 | 0 | 0 | 0 | 0 | 0 | 0 | 0 | 0 | 2 | 0 | 0 | 0 | 0 |
| AE-box | 0 | 0 | 0 | 0 | 0 | 0 | 0 | 0 | 0 | 0 | 0 | 1 | 0 | 0 | 0 | 0 | 0 | 0 | 1 | 1 | 0 | 0 | 0 | 0 |
| GC-motif | 0 | 0 | 0 | 1 | 1 | 3 | 0 | 0 | 0 | 0 | 0 | 0 | 1 | 1 | 2 | 0 | 0 | 0 | 0 | 0 | 1 | 0 | 1 | 1 |
| chs-CMA2a | 0 | 0 | 0 | 0 | 0 | 0 | 0 | 0 | 0 | 0 | 1 | 0 | 0 | 0 | 0 | 0 | 0 | 0 | 1 | 0 | 0 | 0 | 0 | 0 |
| ACE | 0 | 0 | 0 | 0 | 0 | 1 | 0 | 0 | 0 | 0 | 0 | 0 | 0 | 0 | 0 | 0 | 0 | 0 | 1 | 0 | 0 | 0 | 1 | 0 |
| WUN-motif | 0 | 0 | 0 | 0 | 0 | 0 | 0 | 0 | 0 | 0 | 0 | 0 | 0 | 0 | 1 | 0 | 0 | 0 | 1 | 0 | 0 | 0 | 0 | 0 |
| AT-rich element | 0 | 0 | 0 | 0 | 0 | 0 | 0 | 0 | 0 | 0 | 0 | 0 | 0 | 0 | 0 | 0 | 0 | 0 | 1 | 0 | 0 | 0 | 0 | 0 |
| GATT-motif | 0 | 0 | 0 | 0 | 0 | 0 | 0 | 0 | 0 | 0 | 0 | 0 | 0 | 0 | 0 | 0 | 0 | 1 | 0 | 0 | 0 | 0 | 1 | 0 |
| 3-AF3 binding site | 0 | 0 | 0 | 0 | 0 | 0 | 0 | 0 | 0 | 0 | 0 | 0 | 0 | 0 | 0 | 0 | 0 | 1 | 0 | 0 | 0 | 0 | 0 | 0 |
| chs-Unit 1 m1 | 0 | 0 | 0 | 0 | 0 | 0 | 0 | 0 | 0 | 0 | 0 | 0 | 0 | 0 | 0 | 0 | 0 | 0 | 0 | 0 | 0 | 0 | 1 | 0 |
| CAG-motif | 0 | 0 | 0 | 0 | 0 | 0 | 0 | 0 | 0 | 0 | 0 | 0 | 0 | 0 | 0 | 0 | 0 | 0 | 0 | 0 | 0 | 1 | 0 | 0 |
| TGA-box | 0 | 0 | 0 | 0 | 1 | 0 | 0 | 0 | 0 | 0 | 0 | 0 | 0 | 0 | 0 | 0 | 0 | 0 | 0 | 0 | 0 | 0 | 0 | 0 |
| LAMP-element | 0 | 0 | 0 | 0 | 0 | 0 | 0 | 0 | 0 | 1 | 0 | 0 | 0 | 0 | 0 | 0 | 0 | 0 | 0 | 0 | 0 | 0 | 0 | 0 |
| GTGGC-motif | 0 | 0 | 0 | 0 | 0 | 0 | 0 | 0 | 0 | 0 | 0 | 1 | 0 | 0 | 0 | 0 | 0 | 0 | 0 | 0 | 0 | 0 | 0 | 0 |

**Supplementary Table 3**. Information of Transcriptome Data in Article

| Name | study title (or SRA study ID) | Variety | Age | Stress | Tissue | DOI |
| --- | --- | --- | --- | --- | --- | --- |
| radicle−Seedling stage | Development | Azhurnaya | Seedling stage | none | radicle | [https://www.science.org/doi/10.1126/science.aar6089](https://www.science.org/doi/10.1126/science.aar6089) |
| stem axis−Seedling stage | Development | Azhurnaya | Seedling stage | none | stem axis | <https://www.science.org/doi/10.1126/science.aar6089> |
| first leaf blade−Seedling stage | Development | Azhurnaya | Seedling stage | none | first leaf blade | <https://www.science.org/doi/10.1126/science.aar6089> |
| roots−three leaf stage | Development | Azhurnaya | three leaf stage | none | roots | <https://www.science.org/doi/10.1126/science.aar6089> |
| third leaf blade−three leaf stage | Development | Azhurnaya | three leaf stage | none | third leaf blade | [https://www.science.org/doi/10.1126/science.aar6089](https://www.science.org/doi/10.1126/science.aar6089) |
| fifth leaf blade−fifth leaf stage | Development | Azhurnaya | fifth leaf stage | none | fifth leaf blade | <https://www.science.org/doi/10.1126/science.aar6089> |
| roots−Tillering stage | Development | Azhurnaya | Tillering stage | none | roots | [https://www.science.org/doi/10.1126/science.aar6089](https://www.science.org/doi/10.1126/science.aar6089) |
| shoot axis−Tillering stage | Development | Azhurnaya | Tillering stage | none | shoot axis | [https://www.science.org/doi/10.1126/science.aar6089](https://www.science.org/doi/10.1126/science.aar6089) |
| first leaf blade−Tillering stage | Development | Azhurnaya | Tillering stage | none | first leaf blade | <https://www.science.org/doi/10.1126/science.aar6089> |
| roots−30% spike | Development | Azhurnaya | 30% spike | none | roots | [https://www.science.org/doi/10.1126/science.aar6089](https://www.science.org/doi/10.1126/science.aar6089) |
| flag leaf blade−30% spike | Development | Azhurnaya | 30% spike | none | flag leaf blade | <https://www.science.org/doi/10.1126/science.aar6089> |
| spike−Full boot | Development | Azhurnaya | Full boot | none | spike | <https://www.science.org/doi/10.1126/science.aar6089> |
| grain_Chinese Spring | ERP016738 | Chinese Spring | milk grain | none | grain | <https://doi.org/10.1101/gr.217117.116> |
| spike_Chinese Spring | CS_spike | Chinese Spring | Ear emergence | none | spike | [https://www.science.org/doi/10.1126/science.aar6089](https://www.science.org/doi/10.1126/science.aar6089) |
| stem_Chinese Spring | ERP016738 | Chinese Spring | milk grain stage | none | stem | <https://www.science.org/doi/10.1126/science.aar6089> |
| leaf_Chinese Spring | PAMP Triggered Imune Response | Chinese Spring | three leaf stage | none | leaf | [https://www.science.org/doi/10.1126/science.aar6089](https://www.science.org/doi/10.1126/science.aar6089) |
| roots_Chinese Spring | Wulff_H000b | Chinese Spring | 14 days | none | roots | <https://www.science.org/doi/10.1126/science.aar6089> |
| seedling_Chinese Spring | ERP016738 | Chinese Spring | 4 days | none | seeding | <https://www.science.org/doi/10.1126/science.aar6089> |
| ck_Bangladesh_leaf_2 | ERP015130 | Bangladesh | grain filling | ck | leaf | https://doi.org/10.1186/s12915-016-0309-7 |
| Magnaporthe oryzae_Bangladesh_leaf_2 | ERP015130 | Bangladesh | grain filling | Magnaporthe oryzae symptomatic | leaf | https://doi.org/10.1186/s12915-016-0309-7 |
| ck_Bangladesh_leaf_5 | ERP015130 | Bangladesh | grain filling | ck | leaf | https://doi.org/10.1186/s12915-016-0309-7 |
| Magnaporthe oryzae_Bangladesh_leaf_5 | ERP015130 | Bangladesh | grain filling | Magnaporthe oryzae symptomatic | leaf | https://doi.org/10.1186/s12915-016-0309-7 |
| ck_Bangladesh_leaf_7 | ERP015130 | Bangladesh | grain filling | ck | leaf | <https://doi.org/10.1186/s12915-016-0309-7> |
| Magnaporthe oryzae_Bangladesh_leaf_7 | ERP015130 | Bangladesh | grain filling | Magnaporthe oryzae symptomatic | leaf | https://doi.org/10.1186/s12915-016-0309-7 |
| ck_Bangladesh_leaf_12 | ERP015130 | Bangladesh | grain filling | ck | leaf | https://doi.org/10.1186/s12915-016-0309-7 |
| Magnaporthe oryzae_Bangladesh_leaf_12 | ERP015130 | Bangladesh | grain filling | Magnaporthe oryzae symptomatic | leaf | https://doi.org/10.1186/s12915-016-0309-7 |
| CK_leaf_7d_N9134 | SRP041017 | N9134 | 7 days | ck | leaf | [https://doi.org/10.1186/1471-2164-15-898](https://doi.org/10.1186/1471-2164-15-898) |
| Powdery_mildew_pathogen_E09_24h_leaf_7d_N9134 | SRP041017 | N9134 | 7 days | Powdery mildew pathogen E09 24 hours | leaf | https://doi.org/10.1186/1471-2164-15-898 |
| Powdery_mildew_pathogen_E09_48h_leaf_7d_N9134 | SRP041017 | N9134 | 7 days | Powdery mildew pathogen E09 48 hours | leaf | <https://doi.org/10.1186/1471-2164-15-898> |
| Powdery_mildew_pathogen_E09_72h_leaf_7d_N9134 | SRP041017 | N9134 | 7 days | Powdery mildew pathogen E09 72 hours | leaf | <https://doi.org/10.1186/1471-2164-15-898> |
| stripe_rust_pathogen_CYR31_24h_leaf_7d_N9134 | SRP041017 | N9134 | 7 days | stripe rust pathogen CYR31 24 hours | leaf | https://doi.org/10.1186/1471-2164-15-898 |
| stripe_rust_pathogen_CYR31_48h_leaf_7d_N9134 | SRP041017 | N9134 | 7 days | stripe rust pathogen CYR31 48 hours | leaf | https://doi.org/10.1186/1471-2164-15-898 |
| stripe_rust_pathogen_CYR31_72h_leaf_7d_N9134 | SRP041017 | N9134 | 7 days | stripe rust pathogen CYR31 72 hours | leaf | https://doi.org/10.1186/1471-2164-15-898 |
| CK_leaf_7 days_TAM107 | SRR1542405 | TAM107 | 1 week | ck | leaf | <https://www.science.org/doi/10.1126/science.aar6089> |
| 1 hour of drought stress_leaf_7 days_TAM107 | SRR1542407 | TAM107 | 1 week | 1 hour of drought stress | leaf | <https://www.science.org/doi/10.1126/science.aar6089> |
| 6 hour of drought stress_leaf_7 days_TAM107 | SRR1542409 | TAM107 | 1 week | 6 hour of drought stress | leaf | <https://www.science.org/doi/10.1126/science.aar6089> |
| 1 hour of heat stress_leaf_7 days_TAM107 | SRR1542411 | TAM107 | 1 week | 1 hour of heat stress | leaf | <https://www.science.org/doi/10.1126/science.aar6089> |
| 6 hour of heat stress_leaf_7 days_TAM107 | SRR1542413 | TAM107 | 1 week | 6 hour of heat stress_ | leaf | <https://www.science.org/doi/10.1126/science.aar6089> |
| 1 hour of drought&heat combined stress_leaf_7 days_TAM107 | SRR1542415 | TAM107 | 1 week | 1 hour of drought&heat | leaf | <https://www.science.org/doi/10.1126/science.aar6089> |
| 6 hour of drought&heat combined stress_leaf_7 days_TAM107 | SRR1542417 | TAM107 | 1 week | 6 hour of drought&heat | leaf | <https://www.science.org/doi/10.1126/science.aar6089> |
| CK_shoots_three leaf stage_Manitou | SRR1460551 | Manitou | three leaf stage | ck | leaf | <https://www.science.org/doi/10.1126/science.aar6089> |
| cold 2 weeks (4C)_shoots_three leaf stage_Manitou | SRP043554 | Manitou | three leaf stage | cold 2 weeks (4C) | leaf | [https://www.science.org/doi/10.1126/science.aar6089](https://www.science.org/doi/10.1126/science.aar6089) |

**Supplementary Table 4.** **The FPKM data of *TaMGT* genes in different tissues and environment**

|  | TaMGT1A | TaMGT1D | TaMGT1B | TaMGT4A | TaMGT4B | TaMGT4D | TaMGT5A | TaMGT5D | TaMGT5B | TaMGT3A.3 | TaMGT3D.3 | TaMGT3B.3 | TaMGT3B.1 | TaMGT3D.1 | TaMGT3A.1 | TaMGT3A.2 | TaMGT3D.2 | TaMGT3B.2 | TaMGT7B | TaMGT7D | TaMGT7A | TaMGT2A | TaMGT2D | TaMGT2B |
| --- | --- | --- | --- | --- | --- | --- | --- | --- | --- | --- | --- | --- | --- | --- | --- | --- | --- | --- | --- | --- | --- | --- | --- | --- |
| radicle-Seedling stage | 3.080903333 | 3.985543333 | 7.91778 | 2.305666667 | 4.45026 | 0.981747333 | 11.3355 | 12.4831 | 15.92003333 | 6.516136667 | 9.647936667 | 11.7327 | 0.065643967 | 0.078641933 | 0.0591264 | 0.186297767 | 0.178922 | 0.2102195 | 5.644273333 | 6.445723333 | 5.39148 | 1.851686667 | 3.64164 | 2.278143333 |
| stem axis-Seedling stage | 2.09307 | 2.869183333 | 4.77606 | 21.72813333 | 23.82936667 | 3.086966667 | 7.189173333 | 7.883006667 | 10.72952667 | 3.815693333 | 5.814016667 | 5.800763333 | 0.099327933 | 0.0640426 | 0.108747 | 0.025631433 | 0.024732567 | 0.008236267 | 4.27797 | 3.18599 | 3.887796667 | 2.807963333 | 4.51381 | 1.904346667 |
| first leaf blade-Seedling stage | 2.726203333 | 3.620463333 | 5.18448 | 25.2496 | 20.98806667 | 4.47853 | 9.925723333 | 7.826396667 | 12.15303333 | 5.17246 | 7.09657 | 9.314386667 | 0.0479363 | 0.0159938 | 0.0173606 | 0.092136767 | 0.329870667 | 0.473686667 | 5.18761 | 5.191956667 | 6.52973 | 4.460506667 | 5.91033 | 2.483056667 |
| roots-three leaf stage | 3.318926667 | 4.276783333 | 7.105673333 | 1.653558 | 3.068927 | 0.960025333 | 9.11896 | 7.53776 | 11.63816667 | 6.26612 | 9.57496 | 9.450056667 | 0.0070603 | 0.007146533 | 0 | 0.417710667 | 0.296002967 | 0.069060967 | 4.497423333 | 3.919013333 | 5.125696667 | 1.17474 | 3.445406667 | 2.45614 |
| third leaf blade-three leaf stage | 1.754341333 | 2.072596667 | 3.271713333 | 23.39503333 | 21.59846667 | 3.902913333 | 8.160576667 | 6.77192 | 9.00507 | 4.011773333 | 5.671693333 | 6.586326667 | 0.041455667 | 0.008023267 | 0.009005267 | 0.038546233 | 0.124467433 | 0.617672333 | 3.359716667 | 3.21697 | 4.937416667 | 2.970176667 | 4.17678 | 1.93212 |
| fifth leaf blade-fifth leaf stage | 1.366723333 | 1.791846667 | 3.15227 | 27.33476667 | 24.26823333 | 7.970043333 | 5.483203333 | 6.529966667 | 10.00016667 | 2.864536667 | 5.05807 | 5.86856 | 0.113397467 | 0.035622333 | 0.029060467 | 0.1305754 | 0.4804292 | 0.570233233 | 3.8963 | 4.169506667 | 4.801063333 | 3.238356667 | 4.659953333 | 1.899233333 |
| roots-Tillering stage | 3.73479 | 4.748823333 | 6.851946667 | 0.696530667 | 1.169125 | 0.192273333 | 9.521933333 | 8.784413333 | 10.77005333 | 4.993696667 | 9.29647 | 8.98488 | 0.0620052 | 0 | 0.073028133 | 0.26645 | 0.205505633 | 0.066835333 | 4.09694 | 3.687733333 | 4.116956667 | 1.058717333 | 3.111653333 | 2.068273333 |
| shoot axis-Tillering stage | 3.483923333 | 4.21713 | 6.24212 | 1.43068 | 2.44504 | 0.521709667 | 10.00134667 | 10.02358667 | 14.88063333 | 5.033223333 | 9.13229 | 7.89105 | 0.0071161 | 0.0049969 | 0 | 0.17964 | 0.132965 | 0.158066 | 3.82317 | 4.423776667 | 5.144896667 | 1.052206667 | 2.175423333 | 1.625493333 |
| first leaf blade-Tillering stage | 4.481006667 | 4.3692 | 8.04368 | 18.57803333 | 23.78053333 | 4.98811 | 11.1133 | 8.786956667 | 17.65216667 | 9.923613333 | 15.2954 | 15.5148 | 0.016867367 | 0.0156733 | 0 | 1.005147 | 1.292499333 | 3.243346667 | 4.3515 | 4.85063 | 6.999083333 | 4.654743333 | 5.82737 | 3.37994 |
| roots-30% spike | 5.022176667 | 5.57491 | 8.465193333 | 0.49654 | 0.920457333 | 0.088724667 | 8.98264 | 7.471733333 | 10.07558333 | 6.66115 | 10.60526667 | 10.28882 | 0 | 0 | 0 | 1.972219 | 1.293080667 | 0.5745677 | 4.29531 | 3.219486667 | 4.377983333 | 0.995286667 | 2.709953333 | 2.243203333 |
| flag leaf blade-30% spike | 3.317543333 | 3.124993333 | 5.076816667 | 39.71753333 | 38.85706667 | 9.198246667 | 7.556696667 | 6.91987 | 10.22185667 | 8.233226667 | 12.28633333 | 14.04816667 | 0.035137867 | 0 | 0 | 5.295073333 | 2.277323333 | 5.986836667 | 5.242563333 | 5.62193 | 7.499323333 | 2.979633333 | 3.483433333 | 3.063256667 |
| spike-Full boot | 4.35037 | 5.931076667 | 6.66222 | 4.97681 | 7.59128 | 0.245526667 | 12.69476667 | 13.5723 | 17.43353333 | 4.720336667 | 6.421626667 | 5.750643333 | 0.364296333 | 0.361881333 | 0.223308333 | 0.0096995 | 0.054106967 | 0.038553233 | 3.67939 | 3.6612 | 4.145963333 | 2.056856667 | 3.546616667 | 2.72457 |
| grain_Chinese Spring | 2.99875 | 4.79224 | 11.8818 | 0.827811 | 0.750444 | 0 | 9.33695 | 14.8057 | 10.9953 | 8.7915 | 7.71072 | 5.70818 | 0.442246 | 0.532317 | 0.0231784 | 0.162239 | 0.0887193 | 0.268189 | 2.52548 | 1.80498 | 2.38069 | 0.248836 | 1.57983 | 2.54411 |
| spike_Chinese Spring | 7.96925 | 7.09317 | 10.0698 | 6.95136 | 7.10726 | 0 | 12.325 | 17.7404 | 22.6461 | 8.41823 | 10.0627 | 6.49277 | 0.364643 | 0.582131 | 0.48807 | 0.0319009 | 0.0437793 | 0.0632659 | 5.96274 | 3.84961 | 5.74791 | 2.6497 | 4.22893 | 3.89369 |
| stem_Chinese Spring | 11.515 | 10.6236 | 11.4448 | 40.1428 | 24.7665 | 0.139475 | 14.0793 | 16.8627 | 23.4461 | 44.8193 | 52.5753 | 24.4174 | 0.0098224 | 0 | 0 | 2.50267 | 0.00919619 | 0.18015 | 4.41295 | 4.03132 | 8.08006 | 1.25116 | 2.32304 | 3.86199 |
| leaf_Chinese Spring | 6.57159 | 5.84011 | 8.30217 | 23.2547 | 20.8685 | 0 | 11.7554 | 11.329 | 24.8848 | 17.1754 | 19.003 | 10.5592 | 0 | 0.0263425 | 0 | 0.0561647 | 0.145166 | 0.510679 | 3.90914 | 3.56773 | 7.11218 | 1.26013 | 2.10813 | 2.9445 |
| roots_Chinese Spring | 6.16299 | 5.13503 | 8.26075 | 0.968442 | 1.84961 | 0 | 31.1063 | 22.4728 | 22.8437 | 5.99657 | 7.38148 | 5.58636 | 0.201742 | 0.0612862 | 0.10546 | 2.46726 | 2.34177 | 1.039 | 4.56946 | 5.59835 | 6.96034 | 0.224249 | 2.05064 | 0.848863 |
| seedling_Chinese Spring | 2.38174 | 4.10669 | 6.22946 | 14.5622 | 12.9313 | 0.0890105 | 8.27514 | 10.9929 | 11.5262 | 7.61295 | 8.96276 | 5.63532 | 0.141319 | 0.306772 | 0.038683 | 0.00905979 | 0.0668414 | 0.00898967 | 5.97698 | 4.88105 | 6.08188 | 2.75174 | 4.05692 | 2.52209 |
| ck_Bangladesh_leaf_2 | 3.154235 | 2.54617 | 4.248375 | 9.718825 | 15.6057 | 0.8898415 | 3.539025 | 3.492905 | 4.249745 | 4.708795 | 5.330695 | 7.260565 | 0.166609 | 0.0535675 | 0 | 8.1185 | 4.691555 | 8.40896 | 0.641472 | 2.0691 | 3.45776 | 1.79577 | 1.7131255 | 2.283705 |
| Magnaporthe oryzae_Bangladesh_leaf_2 | 3.754755 | 3.387075 | 5.702485 | 4.529605 | 6.34794 | 1.529715 | 6.802725 | 10.81745 | 8.92804 | 10.4875 | 13.85395 | 27.2406 | 0.591818 | 0.324468 | 0 | 11.42465 | 8.506655 | 12.19425 | 2.35161 | 3.150675 | 4.70054 | 2.510605 | 2.39919 | 3.08104 |
| ck_Bangladesh_leaf_5 | 3.740675 | 5.04717 | 9.400155 | 3.343325 | 3.12635 | 0.2648625 | 12.6339 | 28.40085 | 27.22565 | 13.87865 | 15.53295 | 29.82655 | 1.0274605 | 1.0708855 | 0.0589 | 23.93065 | 20.67375 | 28.88715 | 3.314655 | 3.73073 | 5.698465 | 2.16593 | 2.03214 | 4.14891 |
| Magnaporthe oryzae_Bangladesh_leaf_5 | 3.22111 | 2.8227 | 5.36804 | 16.88245 | 15.16885 | 1.857245 | 6.48018 | 6.577465 | 10.339195 | 7.113565 | 8.045735 | 12.05925 | 0.319196 | 0.3079765 | 0 | 12.9323 | 6.185605 | 12.0115 | 3.90801 | 4.491045 | 5.78831 | 3.13284 | 3.39831 | 3.078415 |
| ck_Bangladesh_leaf_7 | 2.715545 | 2.336415 | 4.04826 | 20.40655 | 17.9284 | 3.52766 | 4.04469 | 7.80949 | 10.46219 | 8.3713 | 8.467055 | 12.61985 | 0.1397935 | 0.11984905 | 0.02412395 | 8.794115 | 3.97945 | 8.812575 | 3.31408 | 3.038065 | 4.43816 | 1.84566 | 3.03589 | 2.810055 |
| Magnaporthe oryzae_Bangladesh_leaf_7 | 2.19253 | 1.668575 | 5.409405 | 2.395045 | 1.36589 | 0.3199275 | 5.58341 | 13.6323 | 12.17785 | 8.611645 | 11.76635 | 25.78005 | 0.3931535 | 1.2258415 | 0 | 13.73335 | 12.8754 | 18.8948 | 2.566565 | 2.360435 | 3.83193 | 0.831446 | 0.878396 | 2.393715 |
| ck_Bangladesh_leaf_12 | 2.998425 | 2.770845 | 4.58143 | 36.1301 | 46.15035 | 5.24326 | 3.212965 | 4.137355 | 6.88822 | 4.997345 | 6.41056 | 11.1528 | 0.0200375 | 0.3099205 | 0.02182175 | 20.9454 | 14.6701 | 20.9564 | 0.9873695 | 3.772745 | 4.11495 | 2.71337 | 3.110935 | 2.27048 |
| Magnaporthe oryzae_Bangladesh_leaf_12 | 2.17223 | 3.07493 | 4.153345 | 15.9045 | 19.09065 | 3.094325 | 2.470905 | 3.503255 | 5.08499 | 5.401055 | 5.576425 | 9.80763 | 0.4489035 | 0.928816 | 0.0273615 | 25.1705 | 17.41545 | 20.9695 | 0.552848 | 3.93556 | 3.800955 | 2.83229 | 4.28158 | 2.500455 |
| CK_leaf_7d_N9134 | 2.752193333 | 2.984996667 | 4.515713333 | 16.6809 | 23.75623333 | 3.45985 | 7.621523333 | 7.777903333 | 14.76653333 | 6.578793333 | 9.06254 | 7.4689 | 0.128386667 | 0.029801567 | 0 | 0.454563767 | 0.0156969 | 0.599879 | 3.57162 | 6.34772 | 11.46506667 | 5.47701 | 6.994173333 | 3.471103333 |
| Powdery_mildew_pathogen_E09_24h_leaf_7d_N9134 | 3.059966667 | 2.87064 | 5.485223333 | 16.97893333 | 19.57213333 | 2.810753333 | 7.21226 | 6.804753333 | 10.5331 | 5.17288 | 6.493896667 | 6.607913333 | 0.16834 | 0.014526233 | 0 | 1.264416667 | 0.0142991 | 0.665675333 | 2.582913333 | 4.33558 | 7.58088 | 2.49618 | 4.12739 | 2.790503333 |
| Powdery_mildew_pathogen_E09_48h_leaf_7d_N9134 | 3.743286667 | 4.78952 | 7.526303333 | 25.08883333 | 31.50553333 | 2.495113333 | 11.63835 | 11.9859 | 22.9005 | 12.80413333 | 15.30679 | 11.94899 | 0.164489333 | 0 | 0.016312533 | 2.967086667 | 0.150748667 | 0.857638 | 3.81286 | 5.361426667 | 10.62887667 | 4.898236667 | 7.06022 | 3.719126667 |
| Powdery_mildew_pathogen_E09_72h_leaf_7d_N9134 | 5.040743333 | 4.675053333 | 8.460216667 | 25.58826667 | 37.94543333 | 3.662183333 | 11.07904 | 10.15869333 | 19.9629 | 12.71711 | 15.67856667 | 10.84537333 | 0.077696833 | 0.017030633 | 0 | 0.670501333 | 0.171405333 | 0.814839667 | 3.901306667 | 6.053783333 | 11.54311333 | 5.625903333 | 6.698843333 | 4.065666667 |
| stripe_rust_pathogen_CYR31_24h_leaf_7d_N9134 | 4.312766667 | 4.232043333 | 9.408903333 | 16.5588 | 10.41811 | 5.85343 | 14.53923333 | 11.30386667 | 16.32033333 | 11.74011333 | 11.7398 | 7.43611 | 0.035205667 | 0 | 0 | 0.402396667 | 0.0166093 | 0.698552333 | 2.318683333 | 5.383053333 | 9.355946667 | 4.4708 | 6.076476667 | 3.782773333 |
| stripe_rust_pathogen_CYR31_48h_leaf_7d_N9134 | 3.645243333 | 2.617803333 | 3.422986667 | 23.96906667 | 26.78873333 | 10.13165667 | 6.66568 | 6.001086667 | 11.61524 | 4.784283333 | 5.156663333 | 6.52057 | 0.0326374 | 0.030140333 | 0.017813933 | 0.701817333 | 0 | 0.360994333 | 3.520626667 | 5.502023333 | 9.564843333 | 5.68929 | 6.319616667 | 2.98993 |
| stripe_rust_pathogen_CYR31_72h_leaf_7d_N9134 | 3.61489 | 3.53319 | 4.590596667 | 23.70173333 | 31.46003333 | 9.174123333 | 9.27991 | 9.794743333 | 16.1537 | 6.76919 | 9.089826667 | 8.87249 | 0.117228333 | 0 | 0 | 0.189472267 | 0.0191557 | 0.802065667 | 2.67827 | 5.11814 | 8.954936667 | 4.36855 | 6.41134 | 3.583373333 |
| CK_leaf_7 days_TAM107 | 2.258535 | 2.527745 | 3.41896 | 6.94205 | 4.524935 | 1.051963 | 5.930765 | 5.289975 | 5.68091 | 5.11834 | 5.61757 | 5.144375 | 0 | 0.0199149 | 0 | 0.719973 | 0.385749 | 1.022867 | 3.494085 | 2.724465 | 4.566995 | 2.282705 | 2.719685 | 1.704165 |
| 1 hour of drought stress_leaf_7 days_TAM107 | 2.77693 | 3.223735 | 3.83296 | 5.97708 | 4.4233 | 0.32141 | 6.6274 | 5.677415 | 7.12599 | 4.993675 | 5.33327 | 4.89892 | 0.04024885 | 0.0871549 | 0.0395385 | 1.377785 | 1.05828 | 2.35336 | 3.44317 | 3.16895 | 4.38444 | 2.058215 | 2.11706 | 1.589625 |
| 6 hour of drought stress_leaf_7 days_TAM107 | 2.378675 | 3.53153 | 5.258595 | 1.043975 | 1.41653 | 0.09322415 | 9.05304 | 7.318185 | 7.78231 | 8.80079 | 9.196775 | 6.74037 | 0.00598485 | 0.413555 | 0 | 0.741016 | 0.5672505 | 1.402915 | 3.09081 | 3.159285 | 3.66603 | 1.1300155 | 1.593145 | 1.44525 |
| 1 hour of heat stress_leaf_7 days_TAM107 | 1.67246 | 1.9986 | 3.418135 | 3.25951 | 3.120125 | 0.4604415 | 3.17676 | 2.64264 | 3.46627 | 4.43043 | 4.671455 | 4.183075 | 0.0314487 | 0 | 0 | 0.3652295 | 0.17726825 | 0.3402275 | 1.769295 | 1.592025 | 2.81721 | 0.9031635 | 1.28998 | 0.5921975 |
| 6 hour of heat stress_leaf_7 days_TAM107 | 2.434395 | 2.82407 | 5.75004 | 4.491385 | 4.790665 | 0.828199 | 9.07663 | 5.521315 | 6.789465 | 14.92105 | 15.58975 | 12.0765 | 0.0059554 | 0.06843005 | 0 | 0.04368755 | 0.0580373 | 0.232299 | 5.40621 | 2.12166 | 4.01246 | 1.65367 | 2.19722 | 3.11321 |
| 1 hour of drought&heat combined stress_leaf_7 days_TAM107 | 1.16487 | 1.658935 | 2.773785 | 3.32223 | 3.01852 | 0.11553735 | 3.56237 | 2.51584 | 3.2517 | 4.607465 | 5.64696 | 4.6819 | 0 | 0.1049525 | 0 | 0.0872374 | 0.0947478 | 0.229898 | 1.9859 | 1.103455 | 1.92264 | 0.475277 | 0.6581345 | 0.725144 |
| 6 hour of drought&heat combined stress_leaf_7 days_TAM107 | 2.078305 | 2.4533 | 3.73358 | 3.080405 | 5.69373 | 0.465913 | 8.83 | 6.82101 | 7.262555 | 11.874 | 13.44605 | 10.14015 | 0 | 0.171853 | 0 | 0.00809685 | 0.05783785 | 0.1649515 | 4.87268 | 1.89299 | 2.676105 | 1.239365 | 1.003739 | 2.157855 |
| CK_shoots_three leaf stage_Manitou | 2.583513333 | 2.258293333 | 3.950733333 | 14.2658 | 17.29123333 | 0.33179 | 7.7951 | 6.857783333 | 10.22587 | 7.897743333 | 9.369273333 | 8.28271 | 0.003847667 | 0.0111711 | 0 | 0.2081 | 0.222053667 | 0.514904667 | 4.303376667 | 2.946816667 | 5.451506667 | 2.99578 | 4.3267 | 2.64805 |
| cold 2 weeks (4C)_shoots_three leaf stage_Manitou | 2.92923 | 2.343773333 | 4.102996667 | 31.87053333 | 43.3785 | 0.582800667 | 20.45196667 | 31.4259 | 33.63826667 | 12.22803333 | 14.7924 | 10.66444667 | 0.047530867 | 0.103958133 | 0 | 0.757906 | 0.709611 | 2.360853333 | 9.222703333 | 8.476216667 | 16.1671 | 5.639993333 | 6.161916667 | 3.619693333 |
